# Myosin 9 and N-glycans jointly regulate human papillomavirus entry

**DOI:** 10.1016/j.jbc.2024.105660

**Published:** 2024-01-17

**Authors:** Yang Zhang, Wei Liu, Fujie He, Yan-Jun Liu, Hao Jiang, Cui Hao, Wei Wang

**Affiliations:** 1 Key Laboratory of Marine Drugs, Chinese Ministry of Education, Shandong Provincial Key Laboratory of Glycoscience and Glycotechnology, School of Medicine and Pharmacy, Ocean University of China, Qingdao, China; 2 Laboratory for Marine Drugs and Bioproducts of Qingdao National Laboratory for Marine Science and Technology, Qingdao, China; 3 Medical Research Center, The Affiliated Hospital of Qingdao University, Qingdao, China; 4 Shanghai Key Laboratory of Medical Epigenetics, International Co-laboratory of Medical Epigenetics and Metabolism (Ministry of Science and Technology), Institutes of Biomedical Sciences, Shanghai Institute of Cardiovascular Diseases, Zhongshan Hospital, Department of Systems Biology for Medicine, Fudan University, Shanghai, China; 5 Sanya Oceanographic Institute, Ocean University of China, Sanya, China

**Keywords:** human papillomavirus, myosin-9, N-glycans, entry receptor, endocytosis pathway

## Abstract

Persistent high-risk HPV infection is closely associated with cervical cancer development, and there is no drug targeting HPV on the market at present, so it is particularly important to understand the interaction mechanism between HPV and the host which may provide the novel strategies for treating HPV diseases. HPV can hijack cell surface heparan sulfate proteoglycans (HSPGs) as primary receptors. However, the secondary entry receptors for HPV remain elusive. We identify myosin-9 (NMHC-IIA) as a host factor that interacts with HPV L1 protein and mediates HPV internalization. Efficient HPV entry required myosin-9 redistribution to the cell surface regulated by HPV-hijacked MEK-MLCK signaling. Myosin-9 maldistribution by ML-7 or ML-9 significantly inhibited HPV pseudoviruses infection *in vitro* and *in vivo*. Meanwhile, N-glycans, especially the galactose chains, may act as the decoy receptors for HPV, which can block the interaction of HPV to myosin-9 and influence the way of HPV infection. Taken together, we identify myosin-9 as a novel functional entry receptor for high-risk HPV both *in vitro* and *in vivo*, and unravel the new roles of myosin-9 and N-glycans in HPV entry, which provides the possibilities for host targets of antiviral drugs.

Human papillomaviruses (HPVs) are a large family of small, non-enveloped DNA viruses comprising more than 100 types (1), which can be classified as “low-risk” or “high-risk” varieties according to their association with cancer (2). Persistent infection of high-risk human papillomavirus (HPV) types 16, 18, 45, and 31 can cause cervical cancer, a significant health burden worldwide (3). Thus, understanding the mechanisms of HPV infection can provide novel strategies for blocking HPV infection (4). HPV infection involves interactions with the endocytic transport machinery, which ultimately facilitates the entry of incoming viral genomes into the trans-Golgi network (TGN) and their subsequent nuclear entry during mitosis. There are many different pathways involved in endocytosis, including clathrin-mediated endocytosis (5), caveolae-mediated endocytosis (6), macropinocytosis (7), and other lipid-sensitive pathways (8, 9, 10). However, there is no generalized mechanism for the mode of HPV endocytosis (11, 12, 13, 14, 15, 16, 17), and the post-endocytic trafficking of HPV required for virus capsid disassembly also remains unclear.

Cell surface glycans such as sialic acids, gangliosides, or heparan sulfate (HS) are exploited by many viruses such as influenza, HSV, SV40, papillomavirus, and other pathogens (18, 19, 20). These glycans primarily serve as the attachment factor for viruses, leading to sequential or parallel engagement of other receptors/coreceptors for cell entry. Many HPV viruses, such as types 5, 11, 16, and 31, have been reported to be able to use cell surface heparan sulfate proteoglycans (HSPGs) as primary receptors or co-receptors in the infection of keratinocytes (21, 22, 23). Although the HSPG is widely accepted as an initial receptor, the secondary receptor for HPV infection has not been entirely determined. The known possible second receptors include EGFR/KGFR, α6/β4 integrin, CD63/CD151, and annexin A2 heterotetramer (15, 24, 25, 26). However, different cells infected by HPV often have different endocytosis pathways, so the functional entry receptors of HPV need to be further elucidated.

Here, we systematically perturbed the function of various endocytic pathways during HPV infection in HeLa, CHOK1, and glycan-deficient CHO mutant cells, using chemical inhibitors, siRNA silencing, and glycosidase treatment. Interestingly, HPV45 and HPV16 infected N-glycans-deficient Lec1 cells through a dynamin and endo-lysosomal system-dependent but actin-independent pathway, which differed from those in HeLa and CHOK1 cells. Notably, the myosin-9 protein was first identified as a functional entry receptor for high-risk HPV in different human cells and mice. Meanwhile, the N-glycans, especially galactose chains, may be the decoy receptors for HPV after initial attachment to GAG chains, which may interfere with myosin-9-dependent HPV entry and infection.

## Results

### N-glycans interfere with HPV infection in human and hamster cells

**Figure 1:**
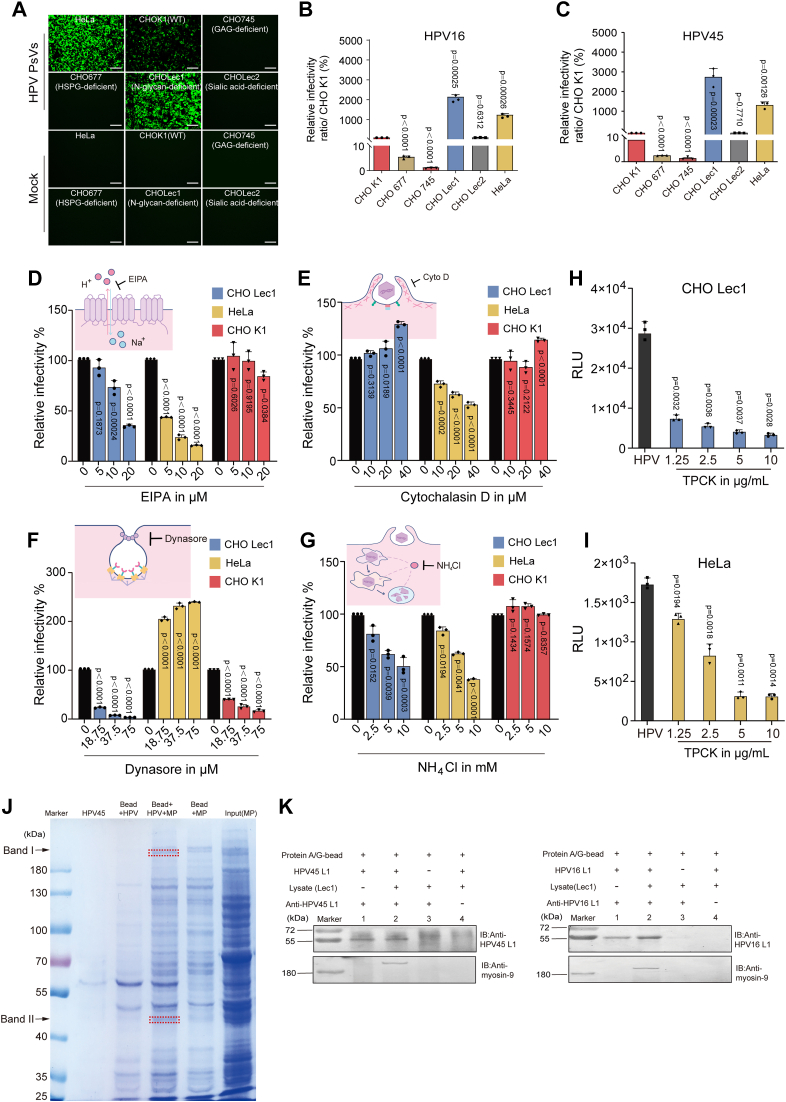
**N-glycans can interfere with HPV infection and identification of a novel HPV entry receptor in HeLa and CHO cells.***A*, the fluorescence levels of infected HPV45 PsVs (5 μl, 10^4^ RLU/μl) in HeLa and different CHO cell lines were evaluated by confocal microscopy at 48 h p.i. Scale bar represents 100 μm. *B* and *C*, HeLa and different CHO cells were infected with HPV16 (*B*) or HPV45 (*C*) PsVs (5 μl, 10^4^ RLU/μl), and the infective levels were evaluated by a luciferase activity assay at 72 h p.i. Each dot represents an individual assay, and values shown are the mean ± SD; *p* values were determined using the one-way ANOVA with Tukey’s post hoc test (n = 3). *p versus* CHOK1 group*. D–G*, CHOLec1, HeLa, and CHOK1 cells were infected with HPV45 PsVs (5 μl,10^4^ RLU/μl) in the presence of the indicated concentrations of EIPA (*D*), Cytochalasin D (*E*), Dynasore (*F*), or NH_4_Cl (*G*) in PBS. Then the infective levels were evaluated by a luciferase activity assay at 48 h p.i. Values are means ± S.D. (n = 3). *p versus* vehicle control group (one-way ANOVA with Tukey's post hoc test). *H* and *I*, CHOLec1 (*H*) and HeLa (*I*) cells were treated with or without TPCK-trypsin at indicated concentrations for 15 min prior to HPV45 PsVs (5 μl, 10^4^ RLU/μl) infection. At 48 h p.i., the infection levels were evaluated by a luciferase activity assay. Values are means ± S.D. (n = 3). *p versus* untreated virus control group (HPV) (one-way ANOVA with Tukey's post hoc test). *J*, an HPV PsVs-based pull-down assay combined with mass-spectrometry was used to identify the entry receptor for HPV45. Bands I and II were identified as NMHC-IIA and actin, respectively. *K*, the cell lysates of Lec1 cells were incubated with HPV45 or HPV16 PsVs (100 μl,10^4^ RLU/μl) for 90 min before incubating with anti-L1 antibody coupled protein A/G magnetic beads for another 90 min, respectively. After washing, the L1 and myosin-9 bound to the beads were analyzed by Western blot.

HPVs have been reported to hijack cell surface HSPGs as primary receptors for the initial binding before entry (27, 28, 29). Recently, Fons and co-workers found that chondroitin sulfate proteoglycans (CSPGs) can function as an alternative initial viral receptor for HPV under high serum conditions (30). However, the relationship between other types of glycans and HPV infection remains elusive. To further explore the relationship between HPV infection and different glycans on the cell surface, the high-risk HPV type 16 and 45 pseudoviruses packaging pCLucf were produced (Fig. S1*A*) and used to infect HeLa, CHOK1, and glycan-deficient CHO cells (CHO677, CHO745, CHOLec1, and CHOLec2). The infection levels of HPV16 and HPV45 PsVs in HeLa and N-glycan-deficient CHOLec1 cells were higher than in other cells according to GFP expression and luciferase activity (Fig. 1, *A*–*C*). Almost no HPV infection was observed in HS-deficient CHO677 and CHO745 cells, consistent with the previous reports that heparan sulfate was indispensable for HPV infection (21, 23). However, the infection levels in Lec1 cells were significantly higher than those in wild-type CHOK1 and sialic acid-deficient Lec2 cells (Fig. 1, *B* and *C*), suggesting that N-glycans may play a negative regulatory role in HPV infection. In addition, no apparent differences on HPV16 and HPV45 infectivity were observed between CHOK1 and CHOPro-5, the parent cell of Lec1 (data not shown), so we used CHOK1 and Lec1 cells in all subsequent experiments.

To test how N-glycans influenced HPV infection, the fluorescently labeled HPV45 particles (AF488 labeled PsVs) were used to track the binding and endocytosis of HPV45 capsids in CHO cells. Notably, more intense fluorescence was observed in Lec1 cells rather than CHOK1 cells at 4 h, 8 h, and 12 h.p.i. (Fig. S1, *B*–*E*), suggesting that the entry efficiency of HPV45 PsVs in Lec1 cells was superior to that in CHOK1 cells. Furthermore, the co-localization signals (yellow dots) between HPV45 L1 and the lysosome marker LAMP1 can be observed at 8 h.p.i. in CHOK1 cells rather than in Lec1 cells (Fig. S2), suggesting that HPV45 PsVs particles may be trafficked to late endosome or lysosome in CHOK1 cells. Interestingly, there was apparent co-localization of HPV45 L1 with ER apparatus rather than TGN region in Lec1 cells at 8 h p.i. (Fig. S2). Thus, N-glycans may influence the entry and intracellular trafficking of HPV in CHO cells.

To further investigate the endocytosis mechanisms of HPV45 in CHO and HeLa cells, we used small molecule inhibitors to perturb the functions of endocytosis pathways commonly used for HPV infection. EIPA, the inhibitor of Na^+^/H^+^ exchanger (17, 31), dose-dependently reduced HPV45 infection in HeLa and Lec1 cells rather than in CHOK1 cells (Fig. 1*D*). The actin-depolymerizing agent cytochalasin D treatment had little effect on HPV45 infection in Lec1 and CHOK1 cells (Fig. 1*E*). Still, dynasore, the inhibitor of dynamin-2 (32, 33, 34), dramatically reduced HPV45 infection in Lec1 and CHOK1 cells rather than in HeLa cells (Fig. 1*F*), suggesting that HPV45 may be internalized by dynamin-dependent but actin-independent endocytosis in CHO cells, different from that in HeLa cells. Similarly, the electron microscopy analysis indicated that many HPV16 PsVs were observed in wider or tubular indentations without visible clathrin-coated pits in HeLa cells, suggesting that HPV16 may enter HeLa cells mainly through macropinocytosis (Fig. S3*A*). Interestingly, NH_4_Cl treatment (35), obviously inhibited HPV infection in Lec1 and HeLa cells rather than in CHOK1 cells (Fig. 1*G*), suggesting that HPV45 may require endosomal acidification for uncoating in Lec1 and HeLa cells rather than in CHOK1 cells. The electron microscopy analysis consistently showed that many HPV particles might be degraded in some giant vesicles in CHOK1 cells rather than HeLa and Lec1 cells (Figs. S3 and S1*F*). Thus, HPV may infect N-glycan deficient Lec1 cells mainly through a dynamin and endo-lysosomal system-dependent entry pathway, different from those in HeLa and CHOK1 cells.

### Myosin-9 may be the novel entry receptor for high-risk HPV in Lec1 and HeLa cells

The apparent differences in the entry pathway and intracellular trafficking of HPV45 among HeLa and CHO cells suggest that a different entry receptor may be in CHO cells for HPV infection. Trypsin can be used to remove proteins from the cell surface, thereby exploring whether viral infection depends on protein receptors. Herein, the trypsin treatment of HeLa and Lec1 cells significantly reduced the infection of HPV45 in a dose-dependent manner (Fig. 1, *H* and *I*), suggesting that some protein receptors may be required for HPV infection in both HeLa and Lec1 cells. Next, we extracted cell membrane proteins and utilized HPV PsVs-based pull-down assay with mass-spectrometry-based proteomics technology to identify the potential HPV entry receptors. We found that myosin-9, a protein of ∼250 kDa (the band I), was pulled down and identified by liquid chromatography-MS/MS (Fig. 1*J*), and the 35 peptide sequences identical to Chinese hamster myosin-9 (NMHC-IIA) are shown in red (Fig. S4*A*). Meanwhile, actin, closely associated with myosin, was identified in band II (Fig. 1*J*), which indicated that membrane-localized actomyosin interacted with HPV PsVs. Furthermore, the coimmunoprecipitation assays verified that NMHC-IIA (myosin-9) can directly interact with the PsVs particles of both HPV45 and HPV16 (Figs. 1*K* and S4*B*), suggesting that myosin-9 may be associated with the entry processes of HPV45 and HPV16.

**Figure 2:**
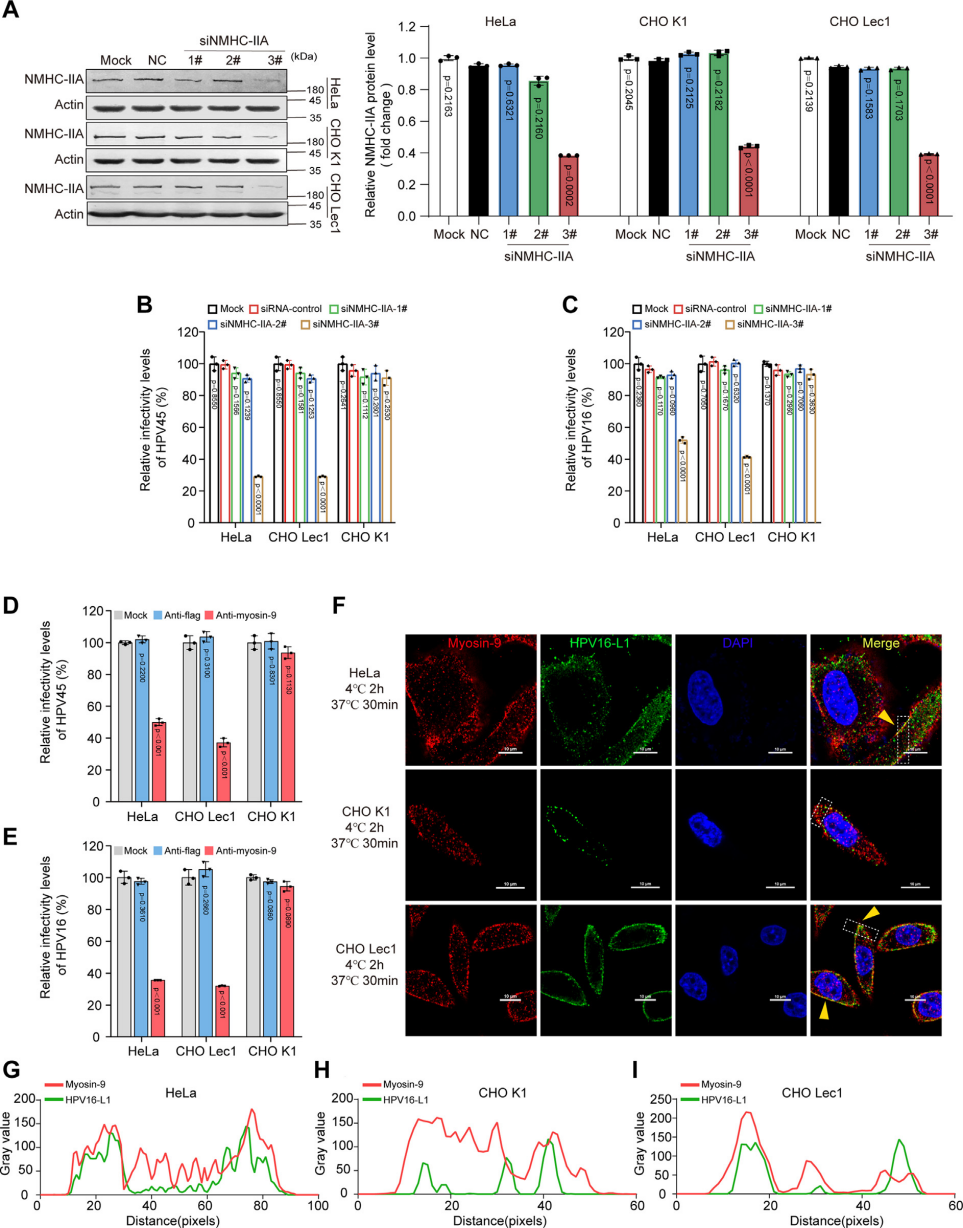
**Myosin 9 is important for HPV infection in HeLa and Lec1 cells.***A*, the effects of siRNAs targeting NMHC-IIA (1#, 2#, 3#) or control siRNA (NC) on NMHC-IIA expression were determined by Western blot 24 h after siRNA transfection. Each dot represents an individual assay, and values shown are the mean ± SD; (n = 3). *p versus* NC group (one-way ANOVA with Tukey's post hoc test). *B* and *C*, the siRNA-treated HeLa, Lec1 or CHOK1 cells were infected with HPV45 (*B*) or HPV16 PsVs (*C*) (5 μl, 10^4^ RLU/μl), and the infective levels were evaluated by a luciferase activity assay at 48 h p.i. Values are means ± S.D. (n = 3). *p versus* siRNA-control group (one-way ANOVA with Tukey's post hoc test). *D* and *E*, HeLa, Lec1, or CHOK1 cells were infected with HPV45 (*D*) or HPV16 (*E*) PsVs (5 μl,10^4^ RLU/μl) in the presence of anti-myosin-9 or anti-flag serum, and the infective levels were evaluated by a luciferase activity assay at 48 h p.i. Values are means ± S.D. (n = 3). *p versus* untreated virus control group (Mock) (one-way ANOVA with Tukey's post hoc test). *F*, HeLa, CHOK1, or Lec1 cells were infected with HPV16 PsVs (100 μl, 10^4^ RLU/μl) at 4 °C for 2 h, followed by 37 °C for 30 min. After that, the co-localization (*yellow arrow*) of L1 and myosin-9 were evaluated by immunofluorescence assay. Scales bars represent 10 μm. *G*–*I*. for co-localization analysis, the fluorescence intensity of the HPV16 L1 and myosin-9 along the line in the square region was calculated by Image J software in HeLa (*G*), CHOK1 (*H*) and CHOLec1 (*I*) cells, respectively.

To evaluate whether myosin-9 contributes to efficient HPV infection in HeLa and CHO cells, the siRNA-based knock-down assays were performed as previously described (36). The results showed that the siRNA 3# markedly reduced the expression of NMHC-IIA in these three cells (Fig. 2*A*). However, the control siRNA (NC) and the other two siRNAs that target NMHC-IIA (1# and 2#) did not significantly reduce NMHC-IIA expression in all three cells (Fig. 2*A*). Then all these cells were infected with HPV45 or HPV16 PsVs. The results indicated that the NMHC-IIA knock-down with siRNA 3# significantly reduced the infection of both HPV45 and HPV16 in HeLa and Lec1 cells (*p* < 0.01), respectively (Fig. 2, *B* and *C*). However, the NMHC-IIA knock-down did not significantly reduce the infection of HPV in CHOK1 cells, suggesting that myosin-9 may be required for HPV infection in HeLa and Lec1 cells rather than in CHOK1 cells (Fig. 2, *B* and *C*). Furthermore, the anti-myosin-9 antibody significantly reduced the infection of HPV45 and HPV16 in HeLa and Lec1 cells rather than in CHOK1 cells, whereas the anti-flag control serum had no inhibition on HPV infection (Fig. 2, *D* and *E*). In addition, the apparent co-localization of HPV16 L1 protein and myosin-9 was found at the cell surface in HeLa and Lec1 cells at 30 min p.i. (yellow linear), while only very few co-localization signals can be found at the cell surface of CHOK1 cells (yellow punctate) (Fig. 2, *F*–*I*). Collectively, these results suggested that the endogenous myosin-9 played a significant role in HPV infection of HeLa and Lec1 cells rather than CHOK1 cells.

### Redistribution of myosin-9 from the cytoplasm to the cell surface is required for HPV entry

**Figure 3:**
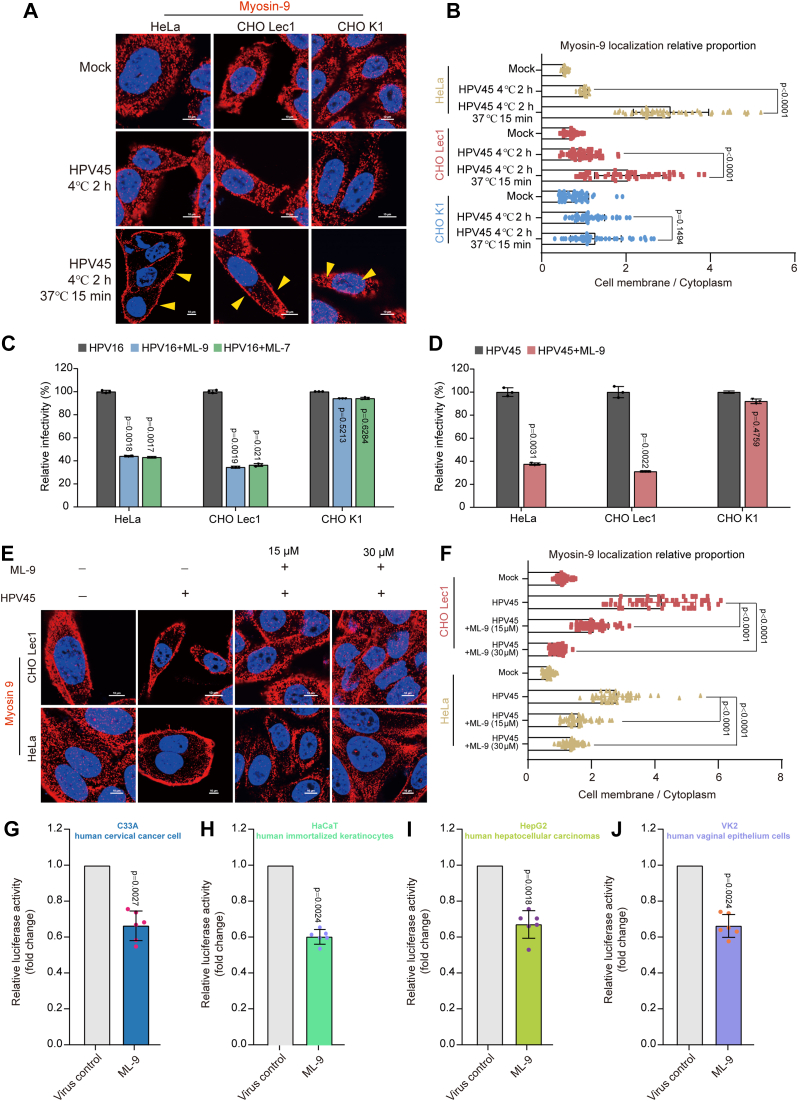
**Redistribution of myosin-9 to the cell surface is required for HPV entry.***A*, HeLa, Lec1, or CHOK1 cells were mock-incubated or exposed to HPV45 PsVs (100 μl, 10^4^ RLU/μl) at 4 °C for 2 h, followed by a temperature shift to 37 °C for 15 min, respectively. Then the localization of myosin-9 was detected by immunofluorescence assay. Scale bars represent 10 μm. *B*, the ratio of myosin-9 localization in the cytoplasm and cell membrane was calculated from HeLa, CHOLec1 and CHOK1 cells by using Image J software. Each dot represents an individual cell, and values shown are the mean ± SD; (n ≥ 50). *p versus* HPV45 4 °C 2 h group (two-sided unpaired *t* test). *C* and *D*, HeLa, Lec1, or CHOK1 cells were infected with HPV16 (*C*) or HPV45 (*D*) PsVs (5 μl, 10^4^ RLU/μl) in the presence or absence of ML-9 (30 μM) or ML-7 (30 μM), respectively. Then the infective levels were evaluated by a luciferase activity assay at 48 h p.i. Values are means ± S.D. (n = 3). *p versus* virus control group (HPV group) *p versus* HPV16 control group (one-way ANOVA with Tukey's post hoc test); *p versus* HPV45 control group (two-sided unpaired *t* test). *E*, Lec1 or HeLa cells were mock-incubated or exposed to HPV45 PsVs (100 μl, 10^4^ RLU/μl) at 4 °C for 2 h, followed by 37 °C for 15 min, in the presence or absence of ML-9 (15, 30 μM). Then the localization of myosin-9 was determined by immunofluorescence assay. Scale bars represent 10 μm. *F*, The ratio of myosin-9 localization in the cytoplasm and cell membrane was calculated from over ten cells by using Image J software. Each dot represents an individual cell, and values shown are the mean ± SD; (n ≥ 50). *p versus* HPV45 group (one-way ANOVA with Tukey's post hoc test). *G*–*J*, C33A (human cervical cancer cell, *G*), HaCaT (human immortalized keratinocytes, *H*), HepG-2 (human hepatocellular carcinomas, *I*), or VK2 (human vaginal epithelium cells, *J*) cells were infected with HPV16 PsVs (5 μl, 10^4^ RLU/μl) in the presence or absence of ML-9 (10 μM), and the infection levels were evaluated by a luciferase activity assay at 48 h p.i. Values are means ± S.D. (n = 6). *p versus* untreated virus control group (two-sided unpaired *t* test).

NMHC-IIA (MYH9) belongs to the myosin superfamily, which has been identified as functional receptors for herpes simplex virus (HSV) (36), Epstein–Barr virus (EBV) (37), Porcine Reproductive and Respiratory Syndrome Virus (PRRSV) (38), and severe acute respiratory syndrome coronavirus 2 (SARS-CoV-2) (39). Herein, we demonstrated that the NMHC-IIA (myosin-9) protein might also function as the HPV entry receptor. Since myosin-9 could translocate from the cytoplasm to the cell surface during HSV entry at 37 °C (36, 40), we further examine whether myosin-9 was redistributed during the initial HPV entry. In cells mock-incubated at 37 °C or exposed to HPV45 only at 4 °C, myosin-9 is mainly localized in the cytoplasm (Fig. 3, *A* and *B*). However, at 15 min after the temperature shift to 37 °C, marked enrichment of myosin-9 at the cell surface was observed in HPV45 infected HeLa and Lec1 cells rather than CHOK1 cells (yellow arrow indicated) (Fig. 3, *A* and *B*). Moreover, apparent enrichment of myosin-9 at the cell surface can also be observed in HPV16 infected HeLa and Lec1 cells rather than CHOK1 cells (Fig. S5). Thus, myosin-9 was redistributed to the cell surface, facilitating initial HPV entry in both HeLa and Lec1 cells.

Redistribution of myosin-9 was reported to be partially regulated by myosin light chain kinase (MLCK) (41). Therefore, we then examined the effects of MLCK inhibitors (ML-9 and ML-7) on the infection of HPV in HeLa and CHO cells. Interestingly, ML-9 or ML-7 (30 μM) treatment significantly reduced the infection of HPV16 in HeLa and Lec1 cells rather than in CHOK1 cells (Fig. 3*C*). ML-9 treatment also significantly reduced the infection of HPV45 in both Lec1 and HeLa cells (*p* < 0.01) (Fig. 3*D*). Consistently, ML-9 (15, 30 μM) treatment also dramatically blocked the enrichment of myosin-9 to the cell surface after HPV45 adsorption at 4 °C followed by a temperature shift to 37 °C in HeLa and Lec1 cells (Fig. 3, *E* and *F*). Thus, the redistribution of myosin-9 from the cytoplasm to the cell membrane was required for efficient HPV entry in HeLa and Lec1 cells rather than in CHOK1 cells. In addition, ML-9 pretreatment also significantly reduced the infection of HPV16 in other human cell lines such as C33A, HaCaT, HepG-2, and VK2 cells as compared to the non-treated control cells (*p* < 0.01) (Fig. 3, *G*–*J*). Taken together, myosin-9 may be involved in the efficient infection of HPV in different human cells.

### The mechanism by which HPV activates myosin-9 *in vitro* and *in vivo*

**Figure 4:**
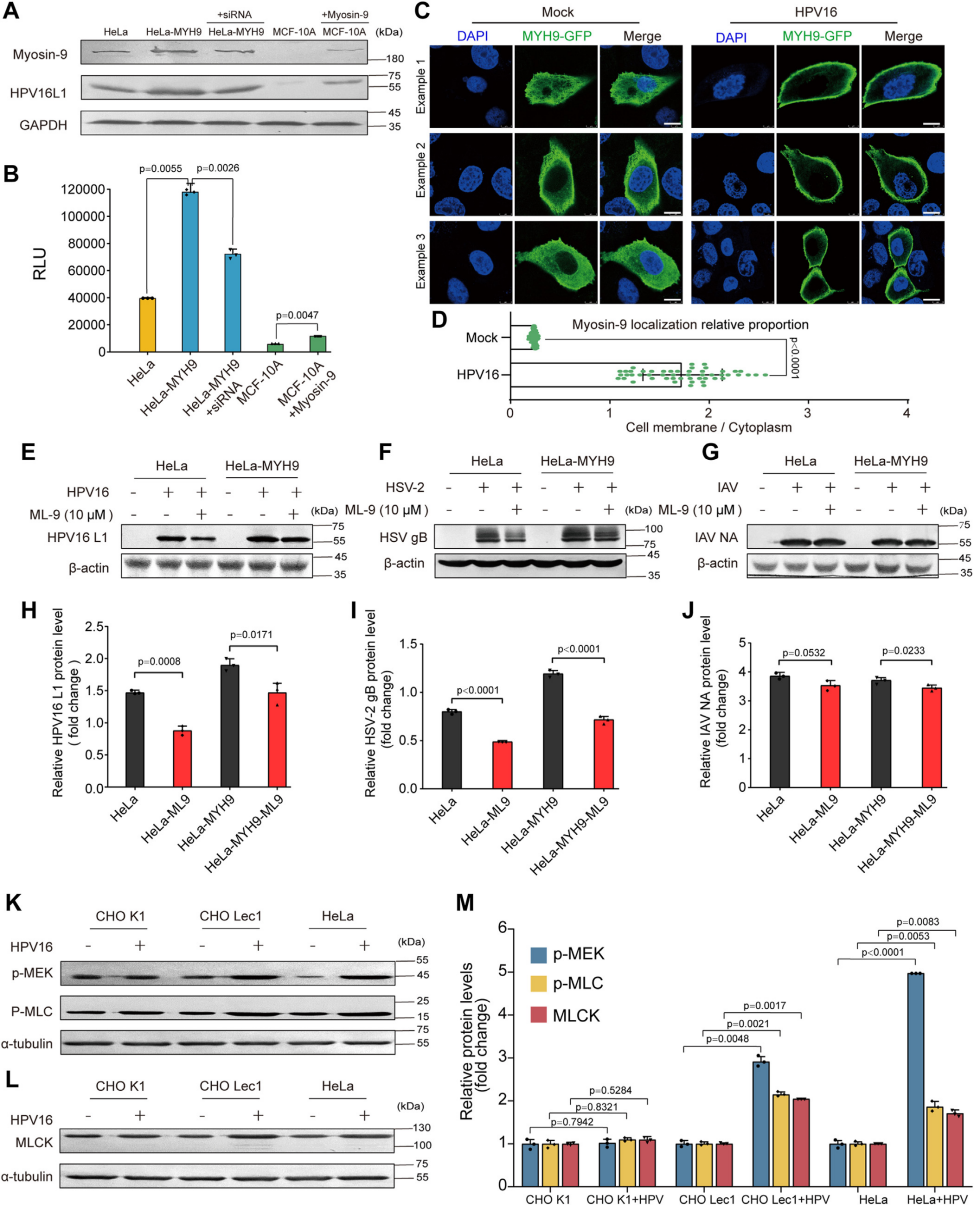
**The molecular mechanism by which HPV activates myosin-9 *in vitro*.***A* and *B*, the expression levels of myosin-9 and HPV16 L1 protein in HeLa cells, HeLa-MYH9 cells with or without myosin-9 siRNAs, and MCF-10A cells with or without myosin-9 overexpression were determined by Western blot 3 h after HPV16 PsVs (100 or 5 μl,10^4^ RLU/μl) infection (*A*). The infective levels were also evaluated by a luciferase activity assay at 48 h p.i (*B*). Values are means ± S.D. (n = 3). *p versus* HPV-infected HeLa-MYH9-WT group or HPV-infected MCF-10A-WT group (one-way ANOVA with Tukey's post hoc test). *C*, the plasmid CMV-GFP-NMHC-IIA transfected MCF-10A cells were mock infected or infected with HPV16 PsVs (100 μl, 10^4^ RLU/μl) at 4 °C for 2 h, followed by 37 °C for 15 min. Then the localization of GFP coupled myosin-9 (MYH-9-GFP) was detected by confocal microscopy. Scale bars represent 10 μm. *D*, the percentage of myosin-9 localization in the cytoplasm and cell membrane was calculated from over ten MCF-10A cells by using Image J software. Each dot represents an individual cell, and values shown are the mean ± SD; (n ≥ 50). *p versus* Mock group (two-sided unpaired *t* test). *E*–*J*, HeLa or HeLa-MYH9 cells were infected with HPV16 PsVs (100 μl,10^4^ RLU/μl) (*E*), HSV-2 (333 strain, MOI = 1.0) (*F*), or IAV (PR8 strain, MOI = 1.0) (*G*) in the presence or absence of ML-9 (10 μM), and the infection levels were evaluated by Western blot at 8 h p.i. Quantification of immunoblot was also shown (*H*–*J*). Values are means ± S.D. (n = 3). *p versus* untreated control group (two-sided unpaired *t* test). *K*–*M*, serum-starved CHOK1, Lec1 or HeLa cells were mock infected or infected with HPV16 PsVs (100 μl,10^4^ RLU/μl) at 4 °C for 2 h, followed by 37 °C for 15 min. Then the levels of the phosphorylated MEK and MLC proteins, or MLCK protein were evaluated by Western blot. Values are means ± S.D. (n = 3). *p versus* non-infected control group (two-sided unpaired *t* test).

Since the activation and redistribution of myosin-9 are required for HPV entry in different human cells, we further explored the mechanism by which HPV activates myosin-9 in different cells. Firstly, we performed a gain-of-function assay by using siRNA of myosin-9 in HeLa-MYH9 cells (stably expressing myosin-9) and overexpression of myosin-9 in MCF-10A cells (nearly no myosin-9 expression) (Figs. 4*A* and S6). As expected, the infection levels of HPV16 in HeLa-MYH9 cells were almost three-fold higher than those in HeLa cells, suggesting that the overexpression of myosin-9 in HeLa cells can enhance the infection of HPV16 (Fig. 4, *A* and *B*). Interestingly, the siRNA of myosin-9 markedly reduced the levels of myosin-9 in HeLa-MYH9 cells. The L1 levels were also lower than those in non-treated HeLa-MYH9 cells (Fig. 4*A*). In contrast, the myosin-9 and L1 levels in myosin-9 overexpressed MCF-10A cells were obviously higher than those in the non-treated MCF-10A cells (Fig. 4*A*). Consistently, the infection levels of HPV16 in HeLa-MYH9 cells could be reduced by using myosin-9 siRNA. The lower infection levels of HPV16 in MCF-10A cells could be reversed by overexpression of myosin-9 (Fig. 4*B*). In addition, the myosin-9-GFP chimeric protein can also be redistributed to the cell surface after HPV16 adsorption at 4 °C followed by a temperature shift to 37 °C in MCF-10A cells (Fig. 4, *C* and *D*), suggesting that HPV16 infection can also activate the heterogenous expressed myosin-9.

Moreover, pretreatment with the myosin-9 inhibitor – ML-9 (10 μM) significantly reduced the infection of HPV16 and HSV-2 rather than the influenza A virus in HeLa cells, suggesting that myosin-9 is required for efficient infection of HPV and HSV rather than IAV (Fig. 4, *E*–*J*). HPV has been reported to use cell surface heparan sulfate proteoglycans (HSPGs) as primary receptors or co-receptors in the infection of keratinocytes (23). The MEK/MLCK pathway is reported to be able to induce the MLC phosphorylation which regulates the redistribution of NMHC-IIA (36). Herein, we also found that the initial infection of HPV16 can significantly increase the levels of MLCK, phosphorylated MEK, and MLC proteins in Lec1 and HeLa cells rather than in CHOK1 cells (Fig. 4, *K*–*M*). Besides that, the treatment of MEK inhibitor Trametinib (15 nM) dramatically inhibited myosin-9 recruitment to the cell surface during HPV initial infection in both HeLa and Lec1 cells (Fig. S7). Taken together, HPV infection may activate the MEK/MLCK/MLC pathway to induce efficient redistribution of myosin-9 to the cell surface.

**Figure 5:**
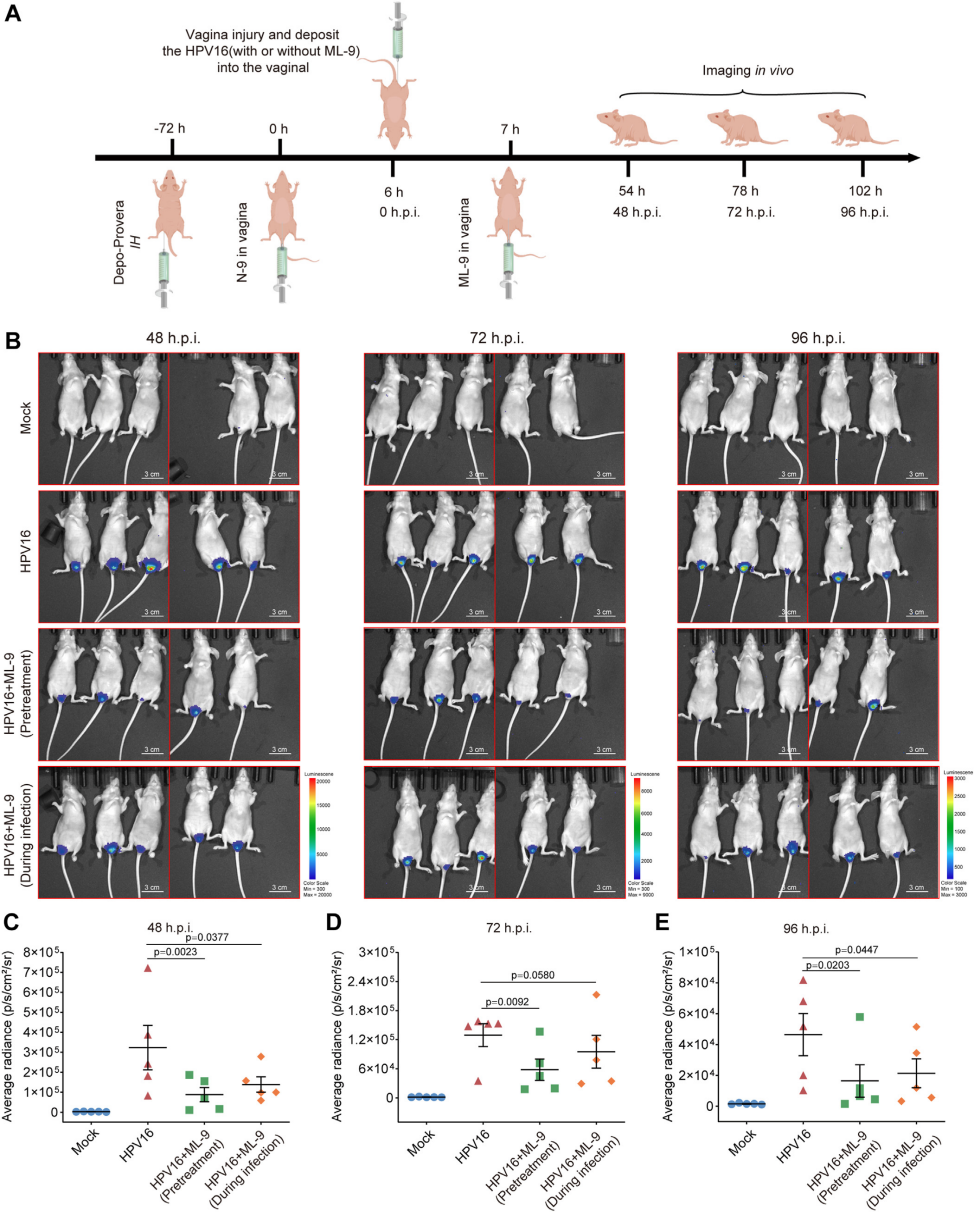
**The involvement of myosin-9 in the infection of HPV16 in mice.** Female BALB/c AnNCr mice were treated with Depo-Provera and nonoxynol-9 to thin and chemically injure the cervicovaginal epithelium before infection with HPV16 PsVs (25 μl, 10^6^ RLU/μl) in the absence or presence of ML-9 (50 μM) (*A*). Then the expression of luciferase was evaluated by performing *in vivo* imaging using an IVIS spectrum imaging system (PerkinElmer, MA, USA) (*B*). *C*–*E*, the *in vivo* images were taken at 48 h p.i. (*C*), 72 h p.i. (*D*) or 96 h p.i. (*E*) were analyzed with the living Image 4.5.2 software to calculate the average radiance (p/sec/cm2/sr). Values are means ± S.D. (n = 5). *p versus* untreated virus control group (HPV16 group) (one-way ANOVA with Tukey's post hoc test).

Furthermore, the role of myosin-9 in HPV infection *in vivo* was also investigated by using an established cervicovaginal challenge model in BALB/c AnNCr mice (Fig. 5). The infection levels of HPV16 PsVs were evaluated by performing *in vivo* imaging using an IVIS spectrum imaging system (PerkinElmer, MA, USA). HPV16 infection induced high luciferase activity in the virus control group (HPV16) compared to the non-infected group (Mock) at 48 h, 72 h, and 96 h.p.i. (Fig. 5, *A* and *B*). However, pretreatment of mouse genital tract with ML-9 before HPV16 inoculation significantly reduced the luciferase expression (Fig. 5, *C*–*E*), suggesting that inhibition of myosin-9 redistribution can block HPV infection in the murine model. Similarly, ML-9 treatment during virus infection also significantly reduced the infection of HPV16 PsVs in the murine model (Fig. 5, *C*–*E*). Thus, ML-9 can also efficiently inhibit HPV infection in mice, suggesting that regulation of myosin-9, including myosin-9 redistribution to the cell surface, was required for efficient HPV infection.

### N-glycans, especially the galactose chains, may be the decoy receptor for HPV

**Figure 6:**
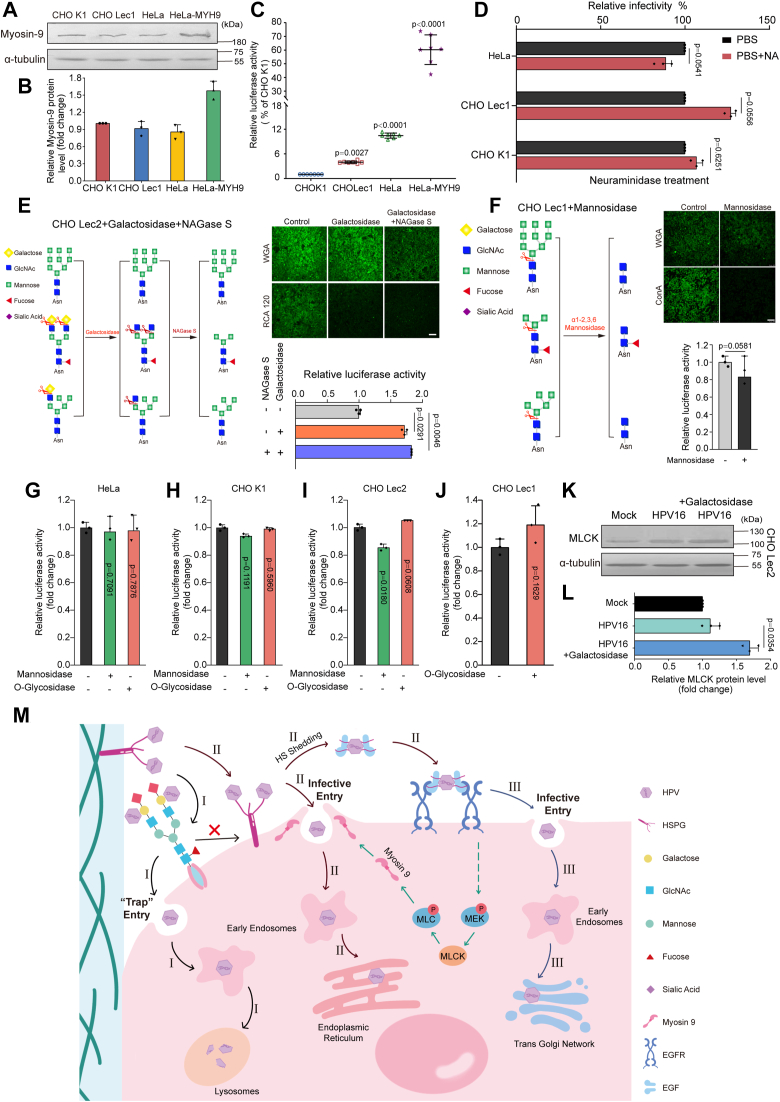
**N-glycans especially the galactose chains may be the decoy receptor for HPV**. *A* and *B*, the expression levels of myosin-9 in CHOK1, Lec1, HeLa and HeLa-MYH9 cells were determined by Western blot. Values are means ± S.D. (n = 3). *C*, CHOK1, Lec1, HeLa, or HeLa-MYH9 cells were infected with HPV16 PsVs (100 μl, 10^4^ RLU/μl). At 72 h p.i., the infective levels were evaluated by a luciferase activity assay. Values are means ± S.D. (n = 7). *p versus* CHOK1 group (one-way ANOVA with Tukey's post hoc test). *D*, HeLa, Lec1, and CHOK1 cells were treated with or without neuraminidase for 1 h before infection with HPV16 PsVs (100 μl, 10^4^ RLU/μl), respectively. The infective levels were evaluated at 48 h p.i. by a luciferase activity assay. Values are means ± S.D. (n = 3). *p versus* PBS group (two-sided unpaired *t* test). *E* and *F*, Lec2 (*E*) or Lec1 (*F*) cells were treated with or without galactosidase, NAGase S, or mannosidase before infection with HPV16 PsVs (5 μl,10^4^ RLU/μl), respectively. The infective levels were evaluated at 48 h p.i. by a luciferase activity assay. Values are means ± S.D. (n = 3). *p versus* untreated virus control group (*E*, one-way ANOVA with Tukey's post hoc test; *F*, two-sided unpaired *t* test). Scale bar represents 100 μm. *G*–*J*, HeLa (*G*), CHOK1 (*H*), Lec2 (*I*), or Lec1 (*J*) cells were treated with or without mannosidase or O-Glycosidase for 1 h before infection with HPV16 PsVs (5 μl,10^4^ RLU/μl), respectively. The infective levels were evaluated at 48 h p.i. by a luciferase activity assay. Values are means ± S.D. (n = 3). *p versus* untreated virus control group (*G*–*I*, one-way ANOVA with Tukey's post hoc test; *J*, two-sided unpaired *t* test). *K* and *L*, serum-starved Lec2 cells were treated with or without galactosidase for 1 h before infection with HPV16 PsVs (100 μl, 10^4^ RLU/μl). Then the expression levels of MLCK protein were evaluated by Western blot. Values are means ± S.D. (n = 3). *p versus* untreated virus control group (HPV16) (two-sided unpaired *t* test). *M*, model for the endocytosis routes of HPV in CHO and HeLa cells. *I*, in CHOK1 cells, HPV may bind to N-glycan chains with galactose, which can induce non-entry receptor mediated endocytosis (“trap” entry). (II) In CHOLec1 and HeLa cells, HPV may initially bind to HSPG, followed by HS shedding, EGF binding, and EGFR activation which may induce the activation of MEK/MLCK/MLC pathway to enhance redistribution of myosin-9 to cell surface, thus inducing myosin-9 mediated endocytosis. (III) In HeLa cells, there may also be another alternative entry pathway for HPV which requires initial HSPG binding, HS shedding, and EGFR mediated macropinocytosis.

To further explore the mechanism by which N-glycans block the redistribution of myosin-9, the expression levels of myosin-9 and the infection ability of HPV16 in CHOK1, Lec1, HeLa, and HeLa-MYH9 were first evaluated (Fig. 6, *A*–*C*). The results indicated that all these cells except HeLa-MYH-9 had nearly the same levels of myosin-9 (Fig. 6*B*), but the infection of HPV16 in CHOK1 cells was significantly lower than those in the other three cells (Fig. 6*C*), suggesting that the N-glycans on CHOK1 cells could interfere with HPV infection without influencing the expression of myosin-9. Moreover, the neuraminidase treatment for 1 h before infection did not increase the infection of HPV16 in CHO and HeLa cells, suggesting that the terminal sialic acid chains on N-glycans did not influence the infection of HPV16 (Fig. 6*D*). Based on this, we then performed HPV16 infection in sialic acid-deficient Lec2 cells in the presence or absence of different glucosidases. Interestingly, the galactosidase and N-acetylgluosaminidase S (NAGase S) treatment significantly increased the infection of HPV16 in Lec2 cells (*p* < 0.05), suggesting that the galactose on N-glycans could interfere with the efficient infection of HPV16 in Lec2 cells (Fig. 6*E*). However, the mannosidase treatment cannot significantly reduce the infection of HPV16 in Lec1 cells, indicating that the mannose chain on N-glycans had little effect on HPV16 infection (Fig. 6*F*). In addition, the O-glycosidase treatment did not significantly influence the infection of HPV16 in HeLa and CHO cells (Fig. 6, *G*–*J*), suggesting that O-glycan was not involved in the infection of HPV16. Thus, N-glycans, especially galactose, acted as the decoy receptor, inhibiting HPV infection.

To further confirm the roles of N-glycans in HPV infection, we determined the influence of galactosidase treatment on MLCK expression in Lec2 cells. The results showed that HPV16 infection did not obviously increase the levels of MLCK in Lec2 cells, but galactosidase treatment before HPV16 infection could significantly increase the levels of MLCK (*p* < 0.05) (Fig. 6, *K* and *L*), suggesting that the galactose chain on N-glycans may inhibit the expression of MLCK to block the redistribution of myosin-9. Furthermore, we also performed the N-glycans overexpression assay on HeLa and Lec1 cells using the plasmids HGNT1 and MGNT1, which encoded N-acetylglucosaminyltransferase. The GnT1 expression was confirmed by the ability of transfected cells to bind FITC-labeled RCA120 (galactose-specific recognition) (Fig. S7*A*, Table S1). After transient transfection of plasmids encoding the human GnT1 gene (HGNT1) or hamster GnT1 gene (MGNT1), the HPV45 infection level significantly decreased in HeLa and Lec1 cells as compared to the non-transfected control cells (Fig. S8, *B* and *C*), respectively, verifying that the increased N-glycan chains may inhibit the infection of HPV in HeLa and CHO cells. Thus, the N-glycans, especially the galactose chains on the cell surface, were the “trap” for HPV infection, which can interfere with activating the MEK/MLCK/MLC pathway to block the efficient redistribution of myosin-9 to the cell surface.

In conclusion, our study identified myosin-9 as a critical host factor for HPV infection and dissected that HPV-hijacked MEK-MLCK signaling regulated the myosin-9 redistribution to the cell surface, facilitating the efficient HPV endocytosis (Fig. 6*M*). Meanwhile, our study proposed that N-glycans, especially the galactose chains on the cell surface, acted as the “trap” for HPV infection, which inhibited MLCK-mediated myosin-9 redistribution and changed the way of HPV infection.

## Discussion

Persistent infection of high-risk HPV may lead to the development of cervical cancers that are a significant health burden worldwide (3). However, there is no generalized mechanism for the mode of HPV endocytosis until now, which restricts the development of novel therapies for HPV infection. In this study, we found that myosin-9 (NMHC-IIA) may be an important functional entry receptor or coreceptor for high-risk HPV both *in vitro* and *in vivo*. Some N-glycan chains especially galactose may be the decoy receptors for HPV which allow the adsorption of HPV but without effective infection (Fig. S1). Interestingly, HPV45 may infect N-glycan deficient CHOLec1 cells through a dynamin-dependent, but actin-independent pathway, which is different from those in HeLa and CHOK1 cells. The viral L1/L2/DNA subcomplex of HPV16 in HeLa cells was reported to be transported to the TGN, Golgi apparatus, and ER (42, 43, 44). Herein, we found that most uncoating HPV45 capsids may be directly trafficked to the ER in Lec1 cells, different from that in HeLa cells. However, the pseudogenome DNA may be largely degraded in lysosomes in CHOK1 cells (Figs. S2 and S3). Thus, N-glycans may influence the endocytosis and intracellular trafficking of both HPV16 and HPV45.

The obvious differences in the entry pathway and intracellular trafficking of HPV in HeLa and Lec1 cells suggest that there may be a different entry receptor for HPV in N-glycan deficient cells. Distinct from the reported possible entry receptors such as EGFR/KGFR, α6/β4 integrin, CD63/CD151, and annexin A2 heterotetramer (15, 24, 25, 45), we first identify that myosin-9 (NMHC-IIA) may be a novel functional entry receptor or coreceptor for HPV16 and HPV45 both *in vitro* and *in vivo*. NMHC-IIA is a subunit of NM-IIA, which is central to the control of cell adhesion, cell migration, and tissue architecture (40). NMIIA often contributes to timely phagocytic internalization and may promote close apposition between the phagocytic cup and the surface of the target (46). Herein, we found that regulation of myosin-9, including myosin-9 redistribution to the cell surface, is required for efficient infection of both HPV16 and HPV45. However, GAG-deficiency sharply decreased the infection of HPV in CHO745 cells (Fig. 1, *B* and *C*), and the reduction of sulfated glycans by chlorate sodium or siRNAs of NDST1 and NDST2 significantly inhibited the infection of HPV in HeLa cells (Fig. S8, *D* and *E*), verifying the critical role of HSPG in the primary adsorption process of HPV. Thus, myosin-9 may function as a secondary entry receptor after initial attachment to GAG chains on the cell surface (Fig. 6*M*), which enables HPV to infect various cell lines and important *in vivo* cellular targets.

Extensive studies have been performed to further explore the roles of N-glycans in myosin-9 activation and HPV entry. Interestingly, the galactosidase treatment significantly increased the infection of HPV16 in Lec2 cells, suggesting that the galactose may interfere with the efficient infection of HPV in CHO cells. Moreover, the initial infection of HPV16 can markedly induce the activation of the MEK/MLCK/MLC pathway in HeLa and N-glycan deficient Lec1 cells rather than in CHOK1 cells, suggesting that the N-glycan chains may interfere with the activation of myosin-9. The activation of MLCK can induce the phosphorylation of MLC, which regulates the redistribution of myosin-9 to the cell surface (36). Besides that, the data of N-glycan profiling indicated that the N-glycans on the surface of HeLa cells have more complex structures and larger molecular weights, while those on CHOK1 cells contain more terminal sialic acid and galactose chains (data not shown). Thus, the specific N-glycans, especially the galactose chains on the cell surface, maybe the decoy receptor for HPV in CHO cells, which may block efficient redistribution of myosin-9 to the cell surface, thus inhibiting the interaction of HPV with the entry receptor – myosin-9 (Fig. 6*M*). In addition, different from CHOK1 cells without EGFR expression, in HeLa cells, there may also be another alternative entry pathway for HPV, which requires EGFR-mediated macropinocytosis (Fig. 6*M*).

## Experimental procedures

### Packaging of the HPV PsVs

HPV16 and HPV45 pseudoviruses (PsVs) were produced according to the previously described methods (45, 47). In brief, 293TT cells were transfected with different HPV capsid plasmids (p16L1L2, p45sheLL), and the reporter plasmid pCLucf at a ratio of 1:1. After 48 h incubation, the cells were then collected and centrifuged using DBPS. The cells were then added with 1.4 volumes of DPBS solution with 9.5 mM MgCl_2_, 1/10 volume of 5% TritonX-100, and 1/500 volume of Plasmid Safe ATP-Dependent DNase, 1/500 volume of Benzonase Nuclease, and 1/50 volume of 1M (NH_4_)_2_SO4 before placed at 37 °C CO_2_-free incubator for 24 h. Then after centrifugation, 1 volume of DPBS solution was added to the vigorously resuspended cell pellet. After that, the cell resuspension was placed in a liquid nitrogen tube to rapidly freeze the solution, then melt at room temperature. Finally, the solution was centrifugated to collect the supernatant followed by the sucrose density gradient ultracentrifugation to obtain the purified HPV pseudovirus.

### Determination of HPV PsVs titer

293FT cells were captured at a density of 1.5 × 10^4^/ml in a 96 well plate and cultured in a 37% and 5% CO_2_ incubator for 16 to 24 h 10 μL of HPV PsVs was added to 293FT cells (with 6 multiple wells), and the 96 well plate was placed in a 37% and 5% CO_2_ incubator for further 48 h. Detect the intensity of luciferase, and the final titer of HPV PsVs is expressed in "RLU/μl". In order to unify the titers of HPV PsVs used in each experiment, we use "RLU/μl" represents the content of HPV PsVs in 293FT cells, and it represents the RLU value that can be detected in 1 μl of HPV PsVs pseudovirus in 293FT cells.

### Identification of proteins that interact with HPV45 PsVs during viral entry

The membrane protein (MP) samples of CHOLec1 cells were first extracted by using ProteoExtract transmembrane protein extraction kit (Merck-Novagen) following its instructions. Then the MP samples were exposed to biotin-labeled HPV45 PsVs at RT for 1 h, and subsequentially immunoprecipitated with streptavidin coupled magnetic beads at RT for another 1 h. Then the immunoprecipitates were separated in a denaturing gel and Coomassie blue stained. Bands detected only in immunoprecipitates from Lec1 cells were excised and digested with trypsin to purify peptides before analysis with a tandem mass spectrometer (LC-MS/MS; Q-Star Elite, Applied Biosystems). The peptide masses obtained by LC-MS/MS analysis were then processed against Chinese hamster protein sequences in the National Center for Biotechnology Information (NCBI) RefSeq database using the Mascot algorithm (Matrix Science). The 35 peptide sequences identical to Chinese hamster myosin-9 (NMHC-IIA) are found.

### Ethics statement

All experiments involving animals were conducted according to the ethical policies and procedures approved by the ethics committee of the School of Medicine and Pharmacy, Ocean University of China (OUCYY-2021001). All methods were performed in accordance with the ARRIVE guidelines.

## Data availability

All data are available within the article or Supporting information.

## Supporting information

This article contains Supporting information.

## Conflict of interest

The authors declare that they have no conflicts of interest with the contents of this article.
