# Supplementary material for: Myosin 9 and N-glycans jointly regulate human papillomavirus entry
Source: J Biol Chem. 2024 Jan 17;300(2):105660. doi: 10.1016/j.jbc.2024.105660 (PMC10865405; doi:10.1016/j.jbc.2024.105660)
Supplement: Supporting Materials, Tables and Figures [file mmc1.pdf]

Supporting Information

Myosin 9 and N-glycans jointly regulate human papillomavirus entry

Yang Zhang[1,2,3,†], Wei Liu[1,4,†], Fujie He[1], Yan-Jun Liu[4], Hao Jiang[1,2], Cui Hao[3,*], Wei Wang[1,2,*]

***Corresponding author:**

Cui Hao: haocui@qduhospital.cn

Wei Wang: wwwakin@ouc.edu.cn

Supplementary Appendix

1. Materials and Methods

2. Supplementary Tables

3. Supplementary Figures

3.1 Figure S1. The influence of N-glycans on the endocytosis of HPV in CHO cells.

3.2 Figure S2. Co-localization patterns of HPV capsid with organelles in CHO cells.

3.3 Figure S3. Ultrastructural morphology of HPV intracellular trafficking in HeLa and CHO cells.

3.4 Figure S4. Interaction between myosin-9 and HPV16 L1 protein.

3.5 Figure S5. Redistribution of myosin-9 to the cell surface is required for HPV16 entry.

3.6 Figure S6. The expression levels of myosin-9 and HPV16 L1 protein in non-infected cells.

3.7 Figure S7. The involvement of MEK in the entry process of HPV16 in HeLa and CHOLec1 cells.

3.8 Figure S8. Profiling the surface glycans of HeLa and CHOLec1 cells with or without GnT1 transfection.

1. Materials and Methods

1.1 Plasmids and cell culture

The plasmids p45sheLL and p16L1L2 encoding HPV capsid proteins and the reporter plasmid pCLucf encoding both firefly luciferase and green fluorescent protein (GFP), were all obtained from Addgene (Cambridge, MA, USA). The plasmid pUC19-hGnT1 encoding human N-acetylglucosaminyltransferase was purchased from Sino Biological (Beijing, China). Myosin-9 expressing plasmid (CMV-GFP-NMHC-IIA) was purchased from Addgene (Cambridge, USA). 293FT and HeLa cells were maintained in Dulbecco's modified Eagle's medium (DMEM, Gibco) containing 10% fetal bovine serum (FBS). CHOK1, CHO677, and CHO745 were maintained in F-12 medium containing 10% FBS. CHOPro-5, CHOLec1, and CHOLec2 cells were maintained in α-minimal essential medium (MEM) containing 10% FBS. HeLa Kyoto cells expressing myosin-9-GFP (HeLa-MYH9) were obtained from Matthieu Piel (kindly provided by Ina Poser), and maintained in DMEM containing 10% FBS and 500 μg/mL G418. MCF-10A cells were maintained in DMEM/F12 containing 5% horse serum (HS), 20 ng/mL EGF, 0.5 μg/mL hydrocortisone, 10 μg/mL insulin, 1% NEAA and 1% ABS. Hep-2, HepG-2, C33A, and VK2 cells were maintained in DMEM containing 10% FBS.

1.2 Production and characterization of HPV pseudovirus (PsVs)

HPV pseudoviruses (PsVs) were produced as previously described[1]. In brief, 293TT cells were transfected with different HPV capsid plasmids (p16L1L2, p45sheLL) and the reporter plasmid pCLucf. After 48 h incubation, cells were harvested and lysed. For optimal maturation, lysates were incubated for a further 24 h with 25 mM ammonium sulfate (pH 9.0)[2]. HPV PsVs were then purified by ultracentrifugation on a 25%-39% linear OptiPrep gradient (Sigma, USA). Virion morphology and quality were visualized by transmission electron microscopy (JEOL JEM-1200EX) as described previously[3]. The infectivity of PsVs was tested by a luciferase assay and GFP detection in 293FT cells.

1.3 Immunofluorescence assay

After being infected with HPV PsVs for different time intervals, cells were washed with PBS and fixed with 4% paraformaldehyde, permeabilized with 0.25% Triton X-100, and blocked with 3% BSA. After that, cells were incubated with anti-HPV L1 antibody (Biodragon Immunotechnologies, Beijing, China) or anti-myosin-9 antibody (Abcam, USA) overnight, followed by FITC labeled or cy3-labeled secondary antibodies (Boster, Wuhan, China) for 60 min. Finally, the cell nuclei were stained with DAPI (Beyotime) for 10 min before being analyzed using a Laser Scanning Confocal Microscope (Zeiss LSM 510 Meta, Germany).

1.4 Western blot assay

Whole-cell protein extracts derived from HPV PsVs-infected cells were subjected to SDS-PAGE and then blotted onto nitrocellulose membranes. After being blocked in Tris-buffered saline (TBS) containing 5% BSA and 0.1% Tween 20 at room temperature (RT) for 2 h, the membranes were washed and incubated at 4 ℃ overnight with primary antibodies against myosin-9 (Abcam, USA), HPV L1 (Biodragon, China), HSV gB (Abcam, USA), IAV NA (Abcam, USA), phosphorylated MEK1/2 (Cell Signaling Technology (CST), Danvers, USA), myosin light chain kinase (MLCK) (HuaBio, Hangzhou, China), phosphorylated myosin light chain (MLC) (HuaBio, China), α-tubulin (CST, USA), GAPDH (CST, USA), or β-actin protein (CST, USA) as a control. The membranes were rinsed and incubated with AP-labeled secondary antibody (1:2000 dilutions; Santa Cruz Biotechnology, USA) at RT for 2 h. The protein bands were then visualized by incubating with developing solution (NBT and BCIP) at RT for 30 min. The relative densities of protein bands were determined using ImageJ software (NIH) v.1.33 u (USA).

1.5 Co-immunoprecipitation assay

For the co-immunoprecipitation assay, the cell lysates of Lec1 cells in the RIPA lysis buffer (1% NP-40, 0.1% deoxycholate, 0.1% SDS, 150 mM NaCl, 1 mM EDTA, and 10 mM Tris-HCl (pH 7.8)) containing a proteinase inhibitor cocktail (GE, USA), were incubated with HPV45 or HPV16 PsVs at RT for 90 min, respectively. Then the anti-HPV45 or anti-HPV16 L1 antibody conjugated protein A/G magnetic beads were added and incubated at RT for another 90 min. After being washed thrice with PBS containing 0.1% Tween 20 (v/v) (PBST), the endogenous myosin-9 bound to the beads was detected by immunoblotting with an anti-myosin-9 antibody. The L1 proteins on the beads were detected by anti-HPV45 or HPV16 L1 antibodies, respectively.

To further verify the direct binding of L1 to myosin-9, the cell lysates (Lys) of 293T cells transfected with the plasmid encoding myosin-9 were first incubated with anti-myosin-9 antibody coupled protein A/G magnetic beads for 90 min at room temperature (RT). After washing the beads with PBS-T for three times, the HPV16 PsVs were added and incubated for another 90 min. After washing thrice with PBST, the L1 and myosin-9 proteins bound to the beads were analyzed by western blot, respectively.

1.6 RNAi experiment

Synthetic small interfering RNA (siRNA) duplexes with symmetrical 3′-deoxythymidine overhangs (Sangon Biotech, Shanghai, China) were used to carry out RNA interference. A series of synthetic siRNA duplexes for the target mRNAs encoding NDST1 and NDST2 were used as follows: NDST1 (Sense: 5′-CCUCCGACUUCUACUUUGAdTdT-3′; Antisense: 5′-UCAAAGUAGAAGUCGGAGGdTdT-3′), NDST2 (Sense 5′-GGACCUUAGUUCCCAACUUdTdT-3′; Antisense 5′-AAGUUGGGAACUAAGGUCCdTdT-3′). Three synthetic siRNA duplexes for the target mRNAs encoding myosin-9 (NMHC-IIA) were also used as follows: 1# (Sense 5’-GGAGCGGAACACUGACCAGTTdTdT-3’; Antisense 5’-CUGGUCAGUGUUCCGCUCCdTdT-3’), 2# (Sense 5’-GAAGAUCAAUCCAUCUUGUdTdT-3’; Antisense 5’-ACAAGAUGGAUUGAUCUUCdTdT-3’), and 3# (Sense 5’-

GCAACAUCGUCUUCAAGAAdTdT-3’; Antisense 5’-UUCUUGAAGACGAUGUUGCdTdT-3’). The control siRNA (NC) was used as follows: Sense: 5’-UUCUCCGAACGUGUCACGUTT-3’; Antisense: 5’-ACGUGACACGUUCGGAGAATT-3’. Briefly, cells were firstly transfected with RNAi oligonucleotides and then washed twice with fresh growth media the next day before infection with HPV45 or HPV16 PsVs. The expression levels of proteins in siRNA-transfected cells were evaluated by western blot 24 hours later.

1.7 Co-localization analysis

CHO cells were infected with HPV45 PsVs for 8 h before performing a double immunofluorescence assay using anti-L1 antibody and anti-TGN46, or anti-LAMP1 antibody (Abcam, USA) followed by FITC- or Cy3-labeled secondary antibodies (Boster, China), respectively. The fluorescence was measured using excitation wavelengths of 488 and 550 nm, respectively, using a confocal laser scanning microscope (Zeiss LSM 710, Germany).

1.8 Electron microscopy

For thin-section EM, HeLa, CHOK1, or Lec1 cells plated onto 35-mm cell culture dishes were incubated with HPV16 PsVs at 4 ℃ for 2 hours before shifted to 37 ℃ for 45 min. After that, cells were scraped off and centrifuged at 1600 × g for 5 min before resuspending with ice-cold fixative (2.5% glutaraldehyde in 0.1 M PBS buffer, pH 7.4) for 40 min. Cells were then pelleted by centrifugation at 20,000 × g for 2 min at RT. Cell pellets were resuspended in 0.5 ml fixative solution then rinsed in 0.5 M cacodylate buffer twice for 10 min and post-fixed with 2% osmium tetroxide for 2 h on ice. The fixed cells were washed with water twice for 10 min, dehydrated with increasing concentrations of ethanol from 50 to 100%, and embedded in spurr resin. Thin (70-80 nm) sections were cut on an ultramicrotome and counter stained with uranyl acetate and lead citrate. The sections were viewed and photographed on a JEM-1200-EX transmission electron microscope (JEOL, Tokyo, Japan) at 80 kV.

1.9 HPV pseudovirus mouse model

Female BALB/c AnNCr mice of six-eight weeks of age were obtained from the Beijing Vital River Laboratory Animal Technology Co., Ltd. (Beijing, China) and raised in a pathogen-free environment (23 ± 2 °C and 55 % ± 5 % humidity). HPV16:pClucf PsVs was used to infect mice as previously described[5,6]. Briefly, female BALB/c AnNCr mice were injected subcutaneously with 3 mg medroxyprogesterone acetate. Three days later, anesthetized mice were treated intravaginally with 20 μL solution containing 4% nonoxynol-9 and 3% carboxyl methyl cellulose (CMC). Six hours later, the anesthetized mice were mock-infected or infected intravaginally with 25 μL HPV16:pCLucf PsVs (1.2×10^{8} IU/mL) suspended in 15 μL 3% CMC after gentle mechanical abrasion with a cytobrush. Some mice also received 40 μL ML-9 (50 μM in 3% CMC) during infection or before infection. At 48 h, 72 h, and 96 h.p.i., the expression of reporter gene luciferase was evaluated by performing *in vivo* imaging using an IVIS spectrum imaging system (PerkinElmer, MA, USA). All images were taken in the IVIS 100 imaging system using medium binning and 300 s exposure and analyzed with the Living Image 4.5.2 software.

1.10 Ethics statement

All experiments involving animals were conducted according to the ethical policies and procedures approved by the ethics committee of School of Medicine and Pharmacy, Ocean University of China (OUCYY-2021001). All methods were performed in accordance with the ARRIVE guidelines.

1.11 Statistical analysis

All data are representative of at least three independent experiments and expressed as the mean ± SD. The significance was analyzed using GraphPad Prism 7.0 (San Diego, USA). Comparisons between groups were performed using two-sided unpaired t test or one-way ANOVA followed by Turkey's test. A statistically significant difference was accessed when $P < 0.05$.

References

1. Buck CB, Thompson CD, Pang Y-YS, Lowy DR, Schiller JT. Maturation of Papillomavirus Capsids. *J Virol*. 2005;79(5):2839-2846. doi:10.1128/jvi.79.5.2839-2846.2005

2. Cardone G, Moyer AL, Cheng N, et al. Maturation of the human papillomavirus 16 capsid. *MBio*. 2014;5(4):1-11. doi:10.1128/mBio.01104-14

3. Cerqueira C, Pang Y-YS, Day PM, et al. A Cell-Free Assembly System for Generating Infectious Human Papillomavirus 16 Capsids Implicates a Size Discrimination Mechanism for Preferential Viral Genome Packaging. *J Virol*. 2016;90(2):1096-1107. doi:10.1128/jvi.02497-15

4. Oyama M, Kozuka-Hata H, Tasaki S, et al. Temporal perturbation of tyrosine phosphoproteome dynamics reveals the system-wide regulatory networks. *Mol Cell Proteomics*. 2009;8(2):226-231. doi:10.1074/mcp.M800186-MCP200

5. Huang HS, Lambert PF. Use of an in vivo animal model for assessing the role of integrin α6B4 and Syndecan-1 in early steps in papillomavirus infection. *Virology*. 2012;433(2):395-400. doi:10.1016/j.virol.2012.08.032

6. Roberts JN, Buck CB, Thompson CD, et al. Genital transmission of HPV in a mouse model is potentiated by nonoxynol-9 and inhibited by carrageenan. *Nat Med*. 2007;13(7):857-861. doi:10.1038/nm1598

2. Supplementary Tables

Table S1. The glycan binding properties of different lectins used in this study.

**Lectin name**	**Common abbreviation**	**Preferred sugar specificity**[a]
Concanavalin A	ConA	αMan, αGlc
Dolichos biflorus	DBA	αGalNAc
Peanut	PNA	Galβ3GalNAc
Ricinus communis I	RCA120	Gal
Soybean	SBA	α>βGalNAc
Wheat Germ	WGA	GlcNAc

[a] **Sugar abbreviations**: Gal, D-Galactose; GalNAc, N-Acetylgalactosamine; Glc, D-Glucose; GlcNAc, N-Acetylglucosamine; Man, Mannose.

3. Supplementary Figures

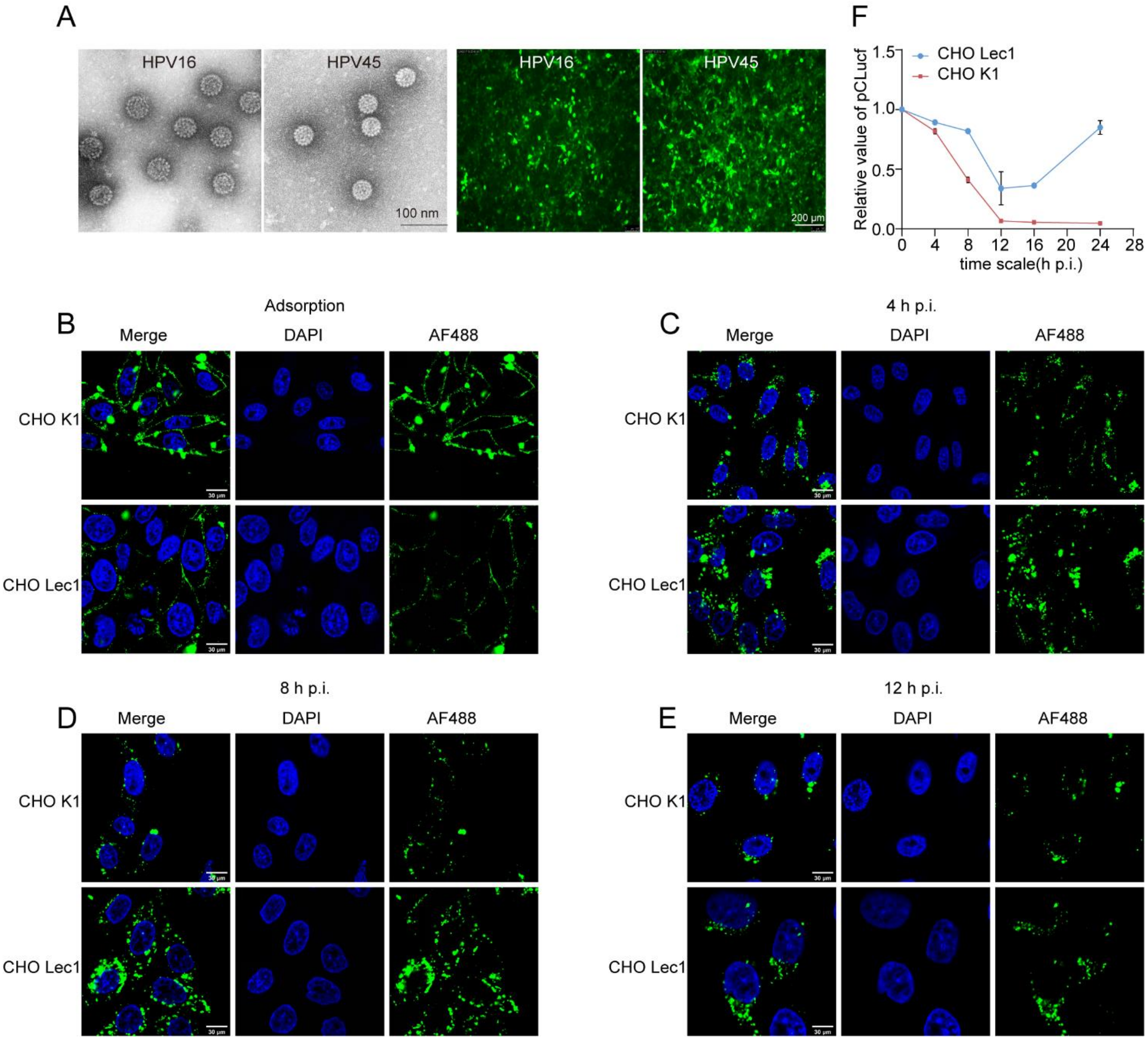

Figure S1. The influence of N-glycans on the endocytosis of HPV in CHO cells. (**A**) Characterization of virion morphology and infectivity ability of the purified HPV45 and HPV16 PsVs. The scale bar represents 100 nm in electron micrographs and 200 μm in fluorescence micrographs. (**B**) CHOK1 and CHOLec1 cells were incubated with Alexa Fluor 488 (AF488) labeled HPV45 PsVs (100 μL,10^4 RLU/μL) at 4 ℃ for 2 h. After washing, the binding amount of HPV45 to the cell surface was detected by a laser scanning confocal microscope (Zeiss LSM 510, Jena, Germany). The scale bar represents 30 μm. (**C-E**) CHOK1 and Lec1 cells were infected with HPV45 PsVs (100 μL,10^4 RLU/μL) at 4 ℃ for 2 h. After washing with PBS, the cells were further incubated at 37 ℃ for 4 h (**C**), 8 h (**D**),

and 12 h (**E**). Then, the localization of HPV45 particles was observed by confocal laser scanning microscopy. Representative images of three independent experiments are shown. The scale bars represent 30 µm. (**F**) CHOK1 and Lec1 cells were infected with HPV45 PsVs (5 µL,10^4 RLU/µL) for the indicated time intervals (4, 8, 12, 16, 20, 24 hours). Then the amount of reporter plasmid DNA was evaluated by a real-time PCR assay. Depicted are the results normalized to the levels of DNA at 0 h p.i. Data represent mean ± S.D. of three independent experiments.

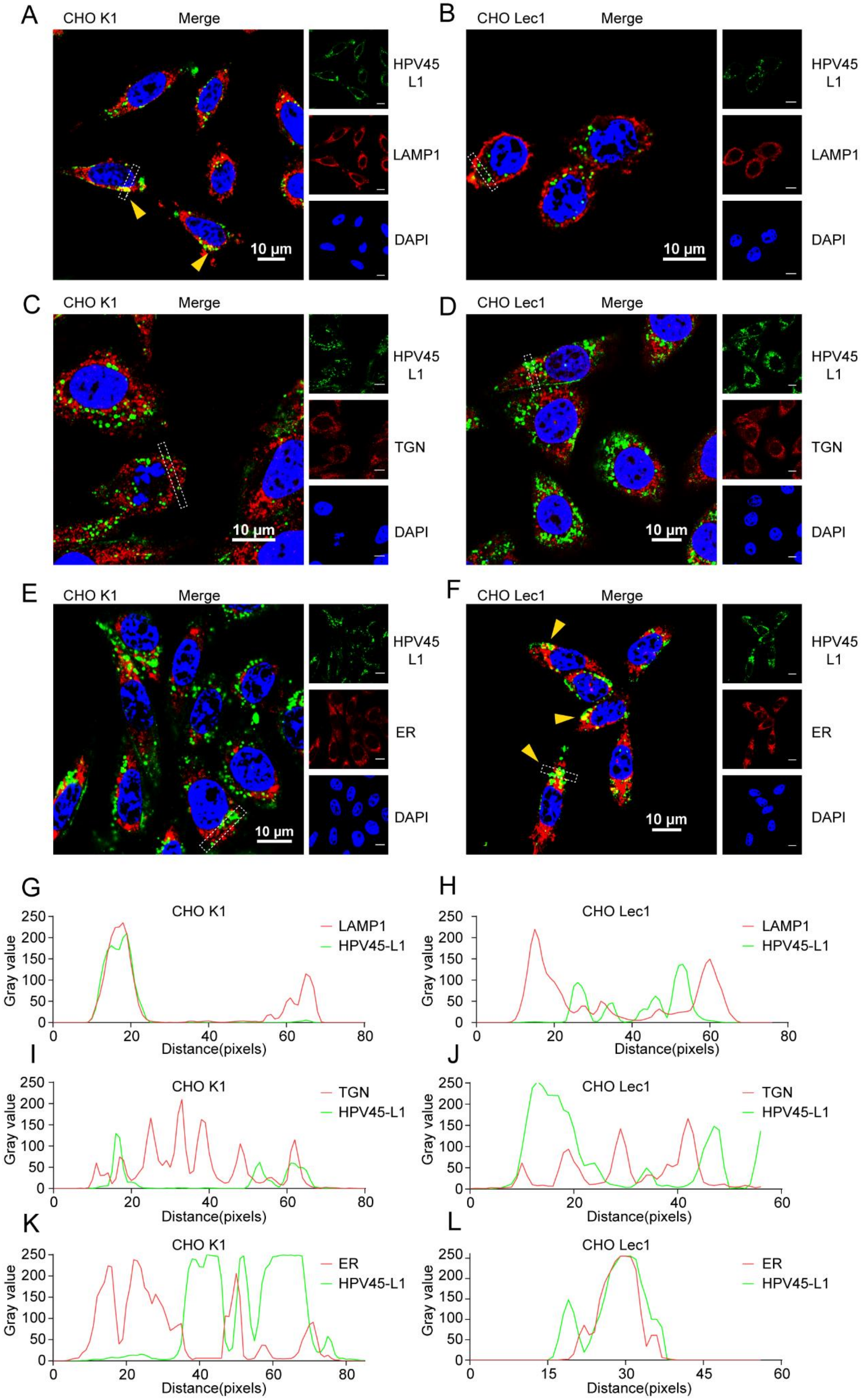

A
CHO K1
Merge
HPV45 L1
LAMP1
DAPI
10 μm
B
CHO Lec1
Merge
HPV45 L1
LAMP1
DAPI
10 μm
C
CHO K1
Merge
HPV45 L1
TGN
DAPI
10 μm
D
CHO Lec1
Merge
HPV45 L1
TGN
DAPI
10 μm
E
CHO K1
Merge
HPV45 L1
ER
DAPI
10 μm
F
CHO Lec1
Merge
HPV45 L1
ER
DAPI
10 μm
G
CHO K1
LAMP1
HPV45-L1
Gray value
Distance(pixels)
H
CHO Lec1
LAMP1
HPV45-L1
Gray value
Distance(pixels)
I
CHO K1
TGN
HPV45-L1
Gray value
Distance(pixels)
J
CHO Lec1
TGN
HPV45-L1
Gray value
Distance(pixels)
K
CHO K1
ER
HPV45-L1
Gray value
Distance(pixels)
L
CHO Lec1
ER
HPV45-L1
Gray value
Distance(pixels)

Figure S2. Co-localization patterns of HPV capsid with organelles in CHO cells. (**A-D**) After infection with HPV45 PsVs (100 μL,10^4 RLU/μL) for 8 h, the localization of HPV L1, LAMP1 (**A and B**), and TGN46 (**C and D**) were determined by immunofluorescence assay, respectively. (**E and F**) CHOK1 and Lec1 cells were infected with HPV45 PsVs (100 μL,10^4 RLU/μL) for 8 h before an immunofluorescence assay of L1. After washing, ER-Tracker Red was added and incubated at 37 °C for 15 min. Finally, the cell nuclei were stained with DAPI before observation under a confocal microscope (Zeiss LSM 510). The scale bar represents 10 μm in the enlarged merge images and the small sized images. (**G-L**) For co-localization analysis, the fluorescence intensity of the L1 protein and organelle signal along the line in the square region was calculated by Image J software in CHOK1 (**G**, **I**, **K**) and CHOLec1 (**H**, **J**, **L**) cells, respectively. The results shown are representative of three independent experiments.

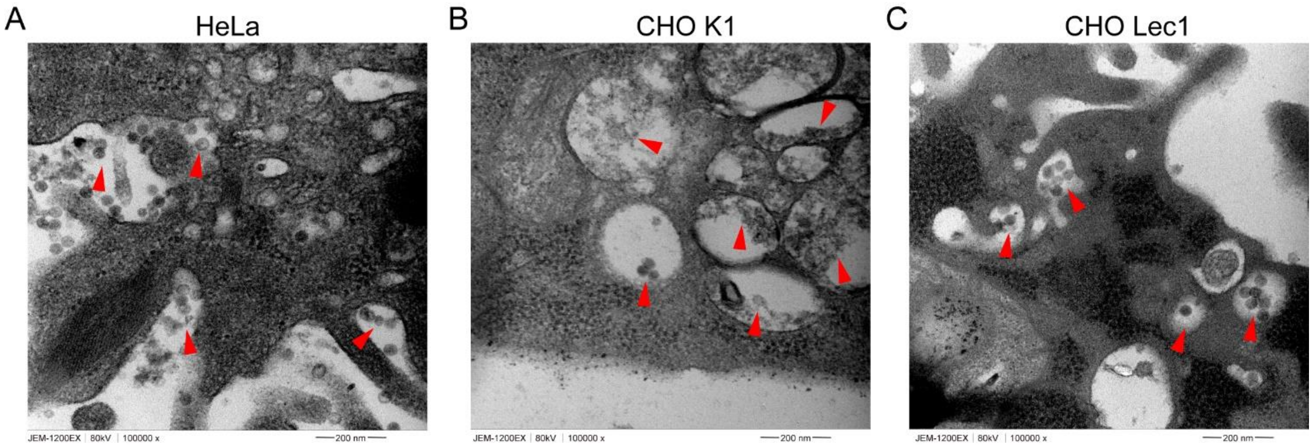

Figure S3. Ultrastructural morphology of HPV intracellular trafficking in HeLa and CHO cells. (**A**-**C**) HeLa, CHOK1, or Lec1 cells were infected with HPV16 PsVs at 4 ℃ for 2 h before shifting to 37 ℃ for 45 min and then fixed by glutaraldehyde. Cells were processed for thin section electron microscopy according to standard procedures. The sections were viewed and photographed on a JEM-1200-EX transmission electron microscope (JEOL, Tokyo, Japan) at 80 kV. Representative images of three independent experiments are shown. Scale bars represent 200 nm. The red arrow indicates the presence of HPV16 PsVs particles.

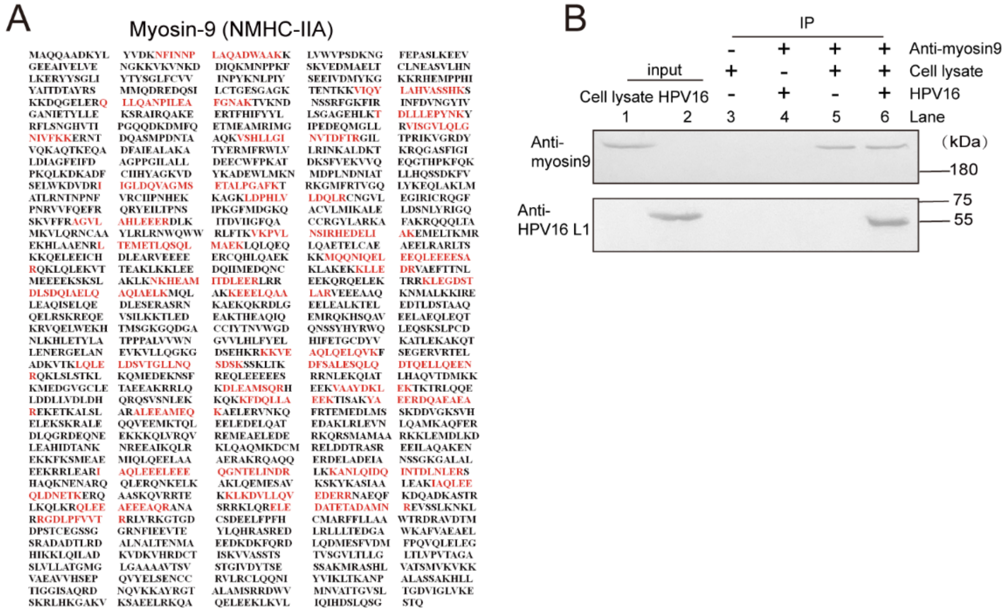

Figure S4. Interaction between myosin-9 and HPV16 L1. (A) Amino acid sequence of Chinese hamster myosin-9 (NMHC-IIA) determined by mass spectrometry analysis. The 35 peptide sequences identical to NMHC-IIA are shown in red. **(B)** The cell lysates (Lys) of 293T cells transfected with the plasmid encoding myosin-9 were incubated with anti-myosin-9 antibody coupled protein A/G magnetic beads for 90 min at room temperature (RT). After washing the beads with PBS-T for three times, the HPV16 PsVs (100 µL,10^4 RLU/µL) were added and incubated for another 90 min. After washing, the L1 and myosin-9 proteins bound to the beads were analyzed by western blot, respectively. The results shown are representative of three independent experiments.

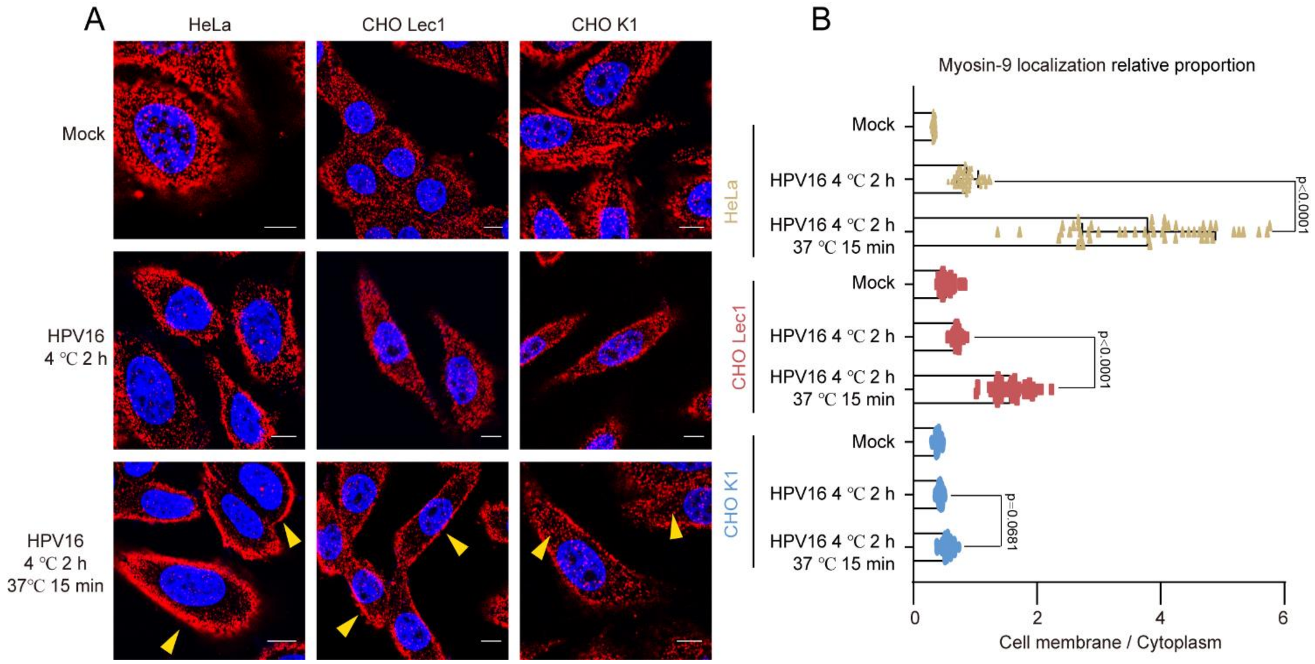

Figure S5. Redistribution of myosin-9 to the cell surface is required for HPV16 entry. (**A**) HeLa, Lec1, or CHOK1 cells were mock-incubated or exposed to HPV16 PsVs (100 μL,10^4 RLU/μL) at 4 ℃ for 2 h, followed by a temperature shift to 37 ℃ for 15 min, respectively. Then the localization of myosin-9 was detected by immunofluorescence assay. Scale bars represent 10 µm. (**B**) The ratio of myosin-9 localization in the cytoplasm and cell membrane was calculated from HeLa, CHOLec1 and CHOK1 cells by using Image J software. Each dot represents an individual mouse, and values shown are the mean ± SD; (n ⩾ 50). *P* vs. HPV16 4 ℃ 2 h group (two-sided unpaired t test).

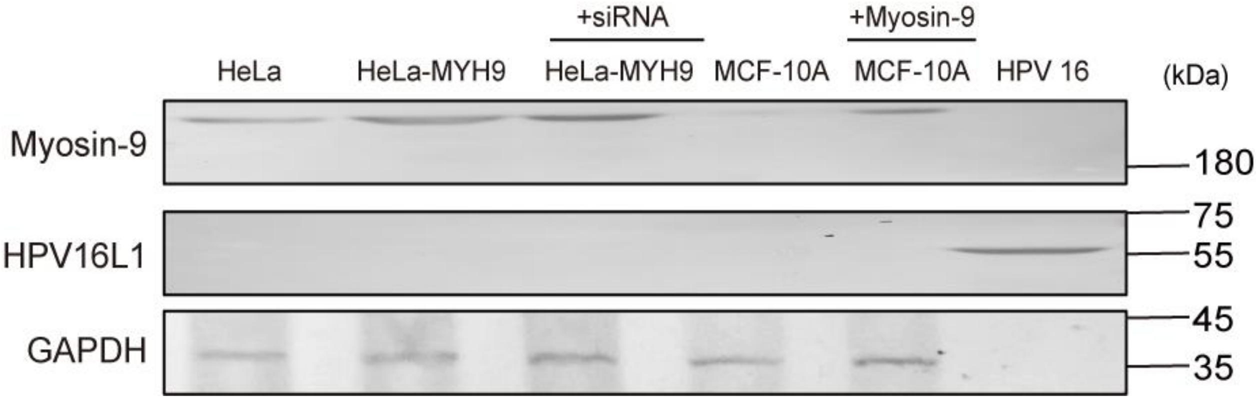

Figure S6. The expression levels of myosin-9 and HPV16 L1 protein in non-infected cells. The expression levels of myosin-9 and HPV16 L1 proteins in the non-infected HeLa cells, HeLa-MYH9 cells with or without myosin-9 siRNAs, and MCF-10A cells with or without myosin-9 overexpression were determined by western blot assay. The HPV16 PsV solution was also included as a positive control for L1 protein. Representative blots of three independent experiments are shown.

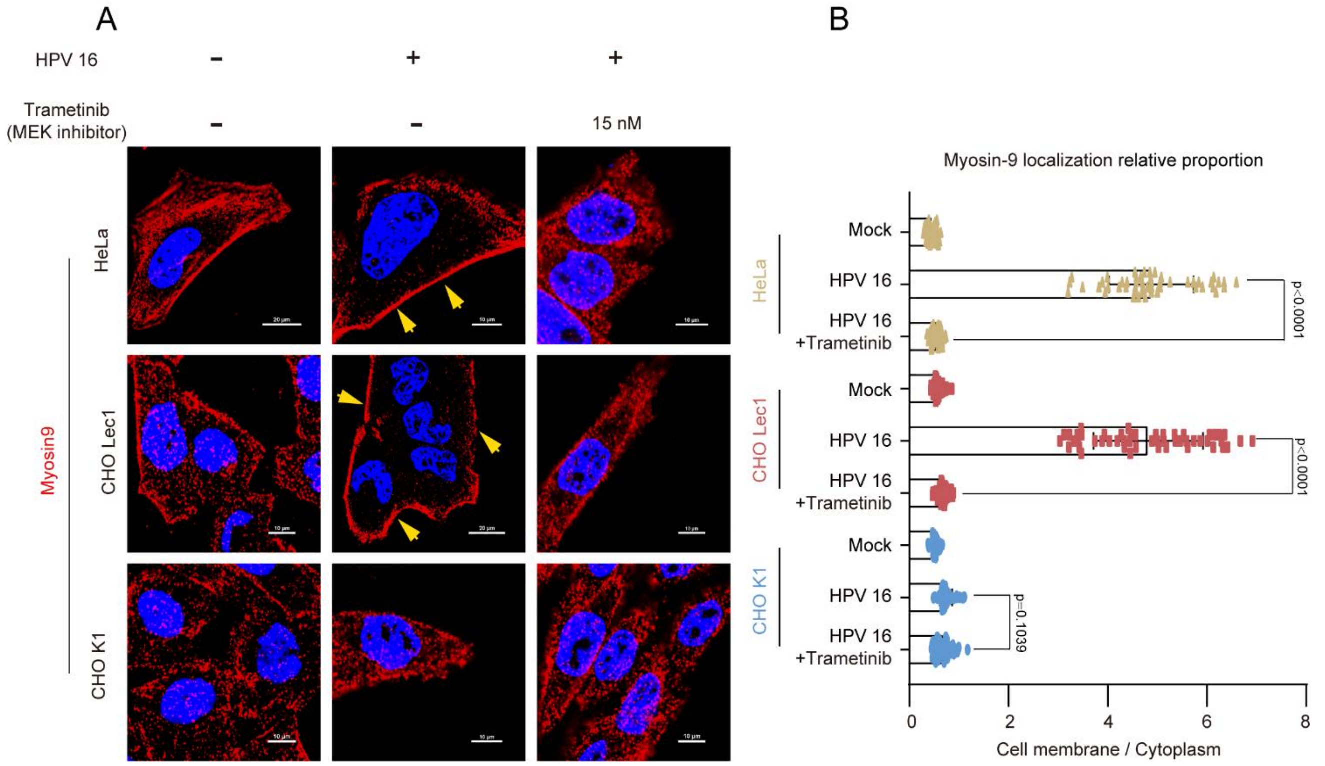

Figure S7. The involvement of MEK in the entry process of HPV16 in HeLa and CHOLec1 cells. (**A**) HeLa, CHOLec1, or CHOK1 cells were mock-incubated or exposed to HPV16 PsVs (100 μL, 10^4 RLU/μL) at 4 ℃ for 2 h, followed by 37 ℃ for 15min, in the presence or absence of Trametinib (15 nM). Then the localization of myosin-9 was determined by immunofluorescence assay. Scale bars represent 10 μm. (**B**) The percentage of myosin-9 localization in the cytoplasm and cell membrane was calculated from over ten cells by using Image J software. Each dot represents an individual mouse, and values shown are the mean ± SD; (n ⩾ 50). *P* vs. HPV16 group (two-sided unpaired t test).

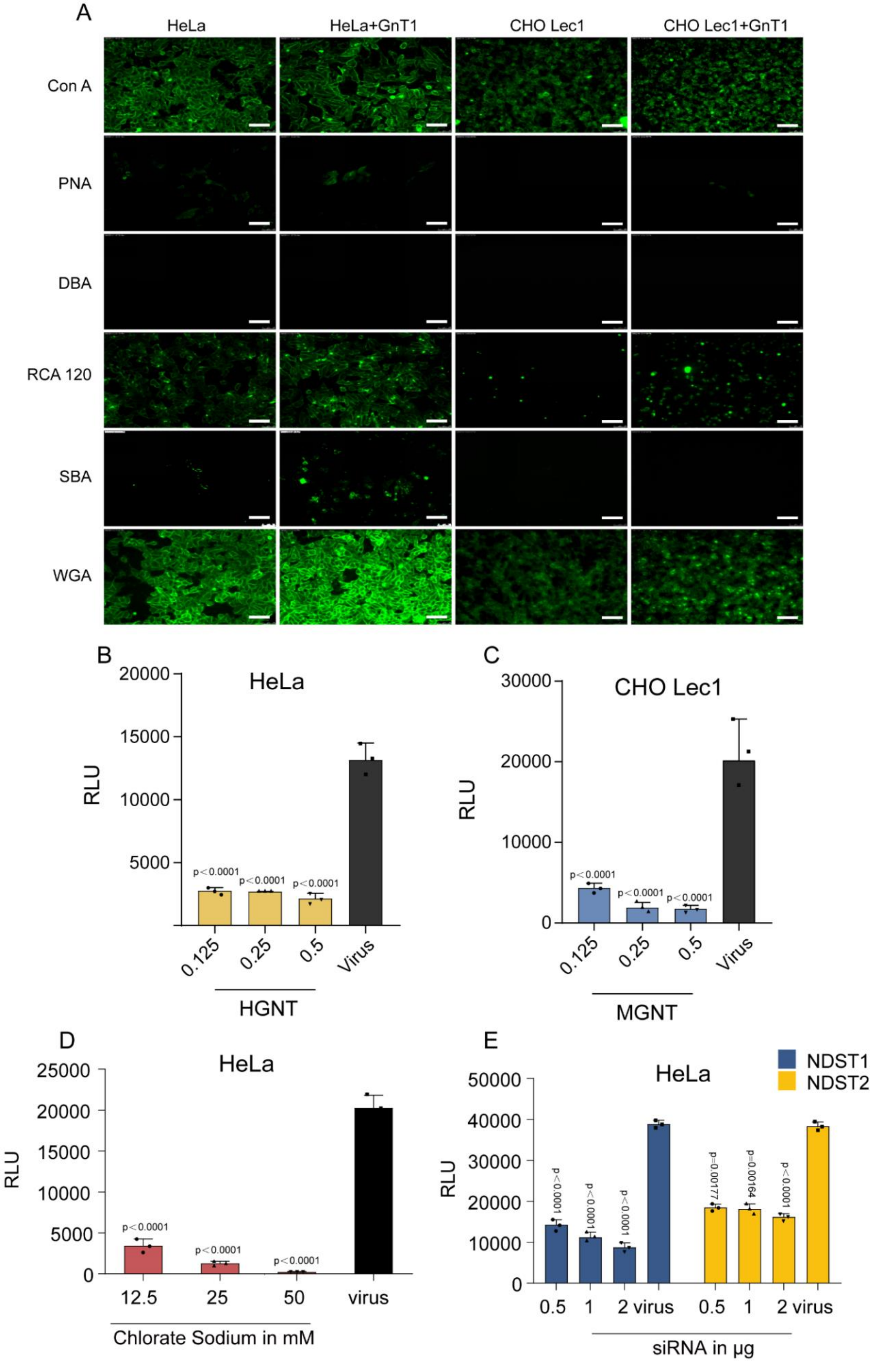

A
HeLa
HeLa+GnT1
CHO Lec1
CHO Lec1+GnT1
Con A
PNA
DBA
RCA 120
SBA
WGA
B
HeLa
RLU
20000
15000
10000
5000
p<0.0001
p<0.0001
p<0.0001
0.125
0.25
0.5
Virus
HGNT
C
CHO Lec1
RLU
30000
20000
10000
0
p<0.0001
p<0.0001
p<0.0001
0.125
0.25
0.5
Virus
MGNT
D
HeLa
RLU
25000
20000
15000
10000
5000
0
p<0.0001
p<0.0001
p<0.0001
12.5
25
50
virus
Chlorate Sodium in mM
E
HeLa
NDST1
NDST2
RLU
50000
40000
30000
20000
10000
0
p<0.0001
p<0.0001
p<0.0001
p=0.00177
p=0.00164
p<0.0001
0.5
1
2 virus
0.5
1
2 virus
siRNA in µg

Figure S8. Profiling the surface glycans of HeLa and CHOLec1 cells with or without GnT1 transfection. (**A**) HeLa and CHOLec1 cells were first transfected with or without plasmids expressing human or hamster N-acetylglucosaminyl transferase 1 (GnT1). After 24 h incubation, the amounts of glycans on the cell surface were determined by using different FITC-conjugated lectins (10 μg/ml, Vector laboratories, USA). Scale bars represent 100 μm. (**B and C**) HeLa (**B**) or Lec1 (**C**) cells were transfected with or without plasmids HGNT1 or MGNT1 at different amounts prior to HPV45 PsVs (5 μL, 10^4 RLU/μL) infection, respectively. At 48 h p.i., the infection levels were determined by measuring the luciferase activity. Values are means ± S.D. (n = 3). *P* vs. virus control group (one-way ANOVA with Tukey's post hoc test). (**D**) HeLa cells were first treated with chlorate sodium in DMEM at different concentrations (12.5, 25, 50 mM) overnight. Then after washing, serum-free DMEM media were added to cells and incubated at 37 °C for 30 min prior to HPV45 PsVs (5 μL, 10^4 RLU/μL) infection. At 48 h p.i., the infection levels were evaluated by measuring the luciferase activity (relative light units, RLU) using the Luciferase Reporter Gene Assay Kit of Beyotime (Nantong, China). Values are means ± S.D. (n = 3). *P* vs. virus control group (one-way ANOVA with Tukey's post hoc test). (**E**) HeLa cells were transfected with or without siRNA oligomers targeting NDST1 or NDST2 at different amounts (0.5, 1.0, 2.0 μg) 24 hours prior to HPV45 PsVs (5 μL, 10^4 RLU/μL) infection. At 48 h p.i., the infection levels were determined by measuring the luciferase activity (relative light units, RLU) and plotted on the Y-axes (error bars represent S.D. derived from triplicate values). The data presented as mean ± S.D. (n = 3). *P* vs. untreated virus control group (one-way ANOVA with Tukey's post hoc test).
